# Glutamine synthetase limits β-catenin–mutated liver cancer growth by maintaining nitrogen homeostasis and suppressing mTORC1

**DOI:** 10.1172/JCI161408

**Published:** 2022-12-15

**Authors:** Weiwei Dai, Jianliang Shen, Junrong Yan, Alex J. Bott, Sara Maimouni, Heineken Q. Daguplo, Yujue Wang, Khoosheh Khayati, Jessie Yanxiang Guo, Lanjing Zhang, Yongbo Wang, Alexander Valvezan, Wen-Xing Ding, Xin Chen, Xiaoyang Su, Shenglan Gao, Wei-Xing Zong

**Affiliations:** 1 Department of Cellular and Genetic Medicine, School of Basic Medical Sciences, Fudan University, Shanghai, China.; 2 Department of Chemical Biology, Ernest Mario School of Pharmacy, Rutgers-The State University of New Jersey, Piscataway, New Jersey, USA.; 3 Rutgers Cancer Institute of New Jersey, Rutgers-The State University of New Jersey, New Brunswick, New Jersey, USA.; 4 Center for Advanced Biotechnology and Medicine, Department of Pharmacology, Robert Wood Johnson Medical School, Rutgers-The State University of New Jersey, Piscataway, New Jersey, USA.; 5 Department of Pharmacology, Toxicology and Therapeutics, The University of Kansas Medical Center, Kansas City, Kansas, USA.; 6 Department of Bioengineering and Therapeutic Sciences and Liver Center, UCSF, San Francisco, California, USA.

**Keywords:** Hepatology, Metabolism, Liver cancer

## Abstract

Glutamine synthetase (GS) catalyzes de novo synthesis of glutamine that facilitates cancer cell growth. In the liver, GS functions next to the urea cycle to remove ammonia waste. As a dysregulated urea cycle is implicated in cancer development, the impact of GS’s ammonia clearance function has not been explored in cancer. Here, we show that oncogenic activation of β-catenin (encoded by *CTNNB1*) led to a decreased urea cycle and elevated ammonia waste burden. While β-catenin induced the expression of GS, which is thought to be cancer promoting, surprisingly, genetic ablation of hepatic GS accelerated the onset of liver tumors in several mouse models that involved β-catenin activation. Mechanistically, GS ablation exacerbated hyperammonemia and facilitated the production of glutamate-derived nonessential amino acids, which subsequently stimulated mechanistic target of rapamycin complex 1 (mTORC1). Pharmacological and genetic inhibition of mTORC1 and glutamic transaminases suppressed tumorigenesis facilitated by GS ablation. While patients with hepatocellular carcinoma, especially those with *CTNNB1* mutations, have an overall defective urea cycle and increased expression of GS, there exists a subset of patients with low GS expression that is associated with mTORC1 hyperactivation. Therefore, GS-mediated ammonia clearance serves as a tumor-suppressing mechanism in livers that harbor β-catenin activation mutations and a compromised urea cycle.

## Graphical abstract

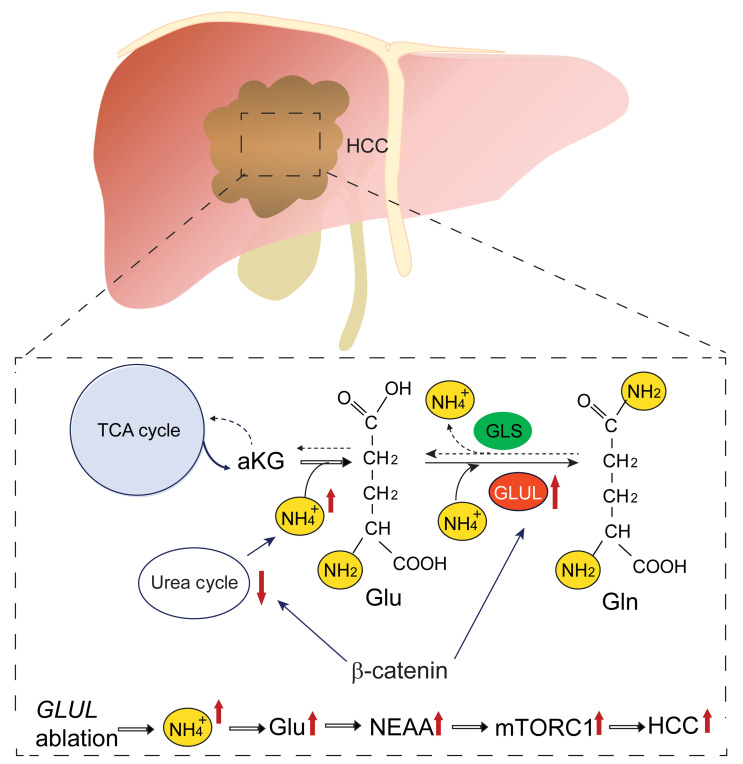

## Introduction

Nitrogen is an essential element in many metabolites, including amino acids, nucleotides, hexosamines, polyamines, and nitric oxides. Mammals obtain nitrogen via dietary supplies, predominantly dietary proteins. Excessive levels of nitrogen waste, mostly produced in the gastrointestinal tracts in the form of ammonia, are cleared in 2 major ways: conversion via the urea cycle into the nontoxic metabolite urea for excretion and assimilation into glutamate (Glu) and subsequently glutamine (Gln). The urea cycle core process is carried out by 5 key urea cycle enzymes (UCEs): carbamoyl-phosphate synthetase 1 (CPS1), ornithine transcarbamylase (OTC), argininosuccinate synthetase (ASS1), argininosuccinate lyase (ASL), and arginase 1 (ARG1). The Glu/Gln nitrogen assimilation process includes 2 steps. The first is the synthesis of Glu from α-ketoglutarate (aKG) and NH_4_^+^, catalyzed by the bidirectional enzyme glutamate dehydrogenase (GDH, or GLUD). The second is the synthesis of Gln by condensing NH_4_^+^ and Glu, catalyzed by glutamine synthetase (GS), encoded by the gene named glutamate ammonium ligase (*GLUL*).

In mammals, the liver and kidney are the organs that handle most ammonia waste. In the liver, hepatocytes are partitioned into 3 metabolic zones along sinusoids between the portal vein and hepatic vein (central vein). UCEs are highly expressed in the periportal and midzonal hepatocytes, and GS expression and activity are high in the pericentral area (1–5). While the urea cycle has been thought to remove the majority of ammonia from the portal blood, GS, which has a high affinity and low *K_m_* for ammonia, removes the residual yet significant amount of ammonia that has escaped the urea cycle. Genetic defects in hepatic UCEs or GS can lead to hyperammonemia, encephalopathy, and even death due to the cytotoxicity of ammonia (6–9), whereas hepatic overexpression of GS ameliorates ammonia toxicity (10).

Hepatocyte partitioning and differential activation of the urea cycle and Gln synthesis are transcriptionally regulated by the Wnt/β-catenin pathway, which is suppressed in the periportal and midzonal areas by high levels of adenomatous polyposis coli expression and is activated in the pericentral zone (11, 12). While the homeostatic level of Wnt/β-catenin signaling is developmentally important, hyperactivation of β-catenin via genetic mutations or upregulation of other oncogenic signaling pathways is a prevalent causative event in hepatocellular carcinoma (HCC). Hyperactive β-catenin signaling has been implicated in regulating the 2 nitrogen-handling pathways. It has been reported that the urea cycle plays a tumor suppressive role, and a defective urea cycle has been found in numerous cancers, including HCC (13, 14). On the other hand, nitrogen assimilation via Glu/Gln biosynthesis is generally thought to be tumor promoting (15, 16). GDH and GS are commonly upregulated in cancers and can promote cell growth by facilitating the biosynthesis of nitrogen-containing metabolites (17–19). The cell autonomous production of Glu and Gln has been suggested to convey cancer cell survival under conditions of Gln auxotrophy in poorly vascularized cancers such as breast cancer and pancreatic ductal carcinoma (PDAC) (15, 18, 19). In the liver, GS was described to enhance HCC development by activating the growth-promoting master anabolic regulator mechanistic target of rapamycin complex 1 (mTORC1) signaling in a mouse model of HCC driven by β-catenin (20), and GS was also shown to play a protumorigenic role in a YAP-driven zebrafish liver cancer model (21). However, the role of GS in HCC development has not been directly tested in mammals. In the current study, we used several mouse models to study the role of GS in HCC and provide surprising evidence that genetic ablation of GS accelerates HCC development.

## Results

### Genetic ablation of Glul leads to increased liver cancer.

We have previously reported that genetic ablation of *Glul*, the gene that encodes GS, in the pancreas leads to decreased PDAC development by breeding the *Glul^fl/fl^* strain (22) with LSL-Kras^G12D/+^ *Trp53^fl/fl^* *Pdx1*-*Cre* mice (15). To study the function of GS in the liver, we bred *Glul^fl/fl^* mice with albumin-Cre (Alb-Cre) mice to specifically knock out GS in hepatocytes and cholangiocytes (Supplemental Figure 1A; supplemental material available online with this article; https://doi.org/10.1172/JCI161408DS1). We did not observe apparent developmental defects between the Alb-Cre^–^ (WT) and Alb-Cre^+^ (*Glul*-KO) mice. Under both fed and fasted conditions, no apparent difference in the expression of Glu/Gln enzymes (GLS1, GLUD1, GOT1, GPT1, IDH2) and UCEs (CPS1, ARG1, OTC) was observed. Sixteen-hour fasting caused similar inhibition of mTORC1 in the WT and *Glul*-KO livers, as measured by phosphorylation of ribosomal protein S6 (S240/244), and similar induction of autophagy as measured by decreased SQSTM/p62 and increased LC3-II (Supplemental Figure 1, A and B). Plasma glucose, insulin, and glucagon levels were also similar between the *Glul*-KO and WT livers (Supplemental Figure 1, C and D). There was no obvious difference in the liver zonation as indicated by IHC staining for ARG1 and CPS1 for the portal vein area (zone 1) and CYP2E1 for the hepatic vein area (zone 3) (Supplemental Figure 1E).

**Figure 1:**
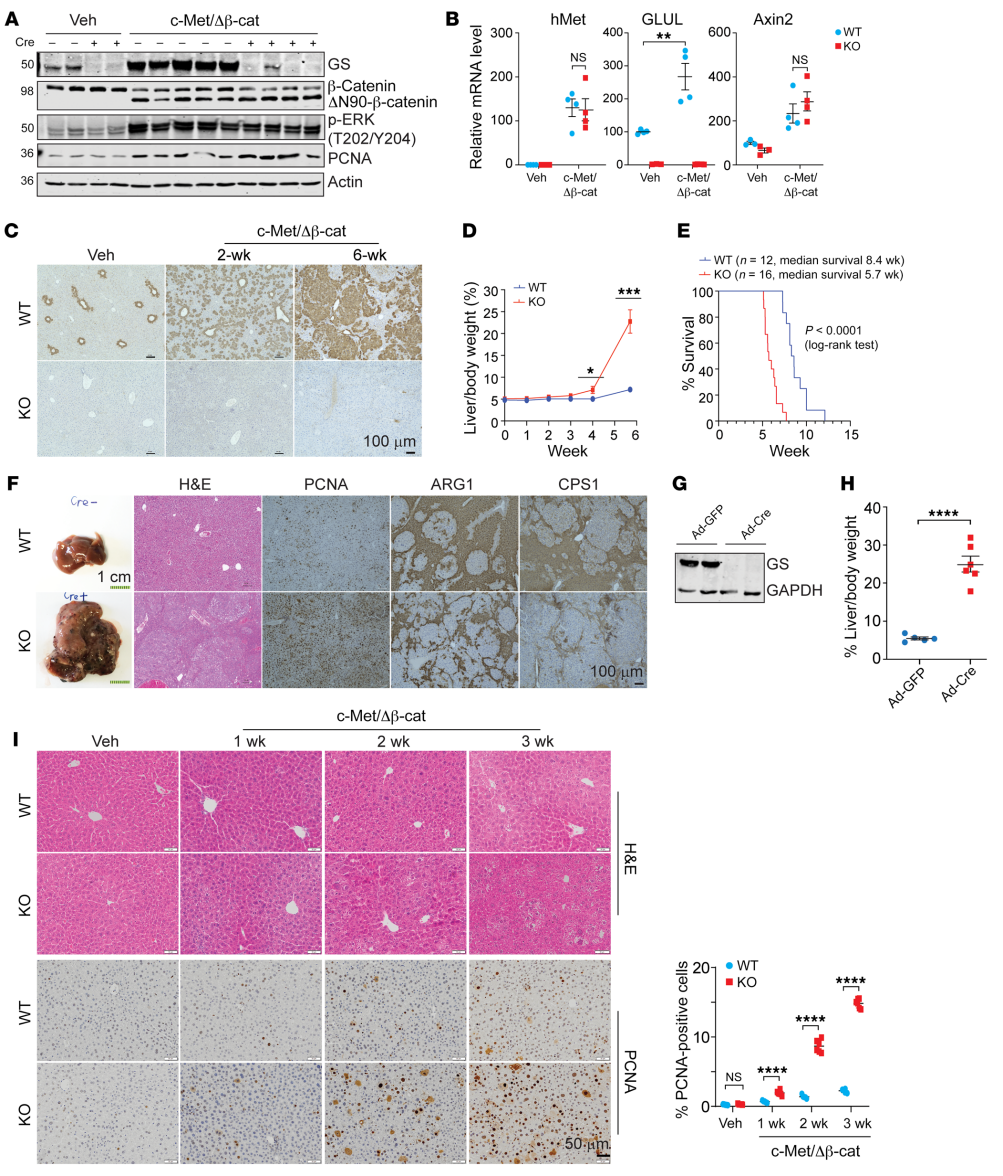
Hepatic ablation of GS exacerbates HCC development driven by c-Met/ΔN90-β-catenin. Seven-week-old *Glul^fl/fl^* Alb-Cre^–^ (WT) and *Glul^fl/fl^* Alb-Cre^+^ (KO) male mice were injected with either vehicle or c-Met/ΔN90-β-catenin/SB10 plasmids via SB-HTVI. Livers were collected and analyzed at the indicated time intervals. (**A**) Immunoblotting of liver tissue samples collected at 6 weeks (endpoint of the KO mice) after HTVI. Representative blots are shown (*n* = 3–5). The protein molecular weight in kDa is indicated on the left. (**B**) Relative mRNA levels in livers 2 weeks after HTVI were determined by qPCR (*n* = 3–4). (**C**) IHC of GS was performed at 0, 2, or 6 weeks after HTVI (*n* = 3). Representative images are shown. (**D**) Liver/body weight ratios were compared (*n* = 3–6). (**E**) Kaplan-Meier curves are shown. (**F**) Representative gross, H&E, and IHC images of liver tissues harvested 6 weeks after oncogene injection (*n* = 3). (**G** and **H**) Seven-week-old *Glul^fl/fl^* male mice were first coinjected with pCMV-c-Met/ΔN90-β-catenin plasmids. One week later, half of the mice were randomly selected and injected with adenoviral CMV-Cre (Ad-Cre), while the other half were injected with adenoviral CMV-GFP (Ad-GFP) as controls via the tail vein. Livers were harvested another 7 days later, and immunoblotting showed successful GS knockout by Ad-Cre (**G**). Mice were harvested at the endpoint (6 weeks after HTVI injection) (*n* = 6). Liver weight/body weight ratios were compared. The results are expressed as mean ± SEM (**H**). (**I**) Liver sections from WT and KO mice were obtained at the indicated time points and processed for H&E and PCNA IHC staining (*n* = 3 mice for each group). Representative images are shown. The number of PCNA-positive cells was quantified by ImageJ from 6 randomly selected fields. Shown on the right is the mean percentage ± SEM. *P < 0.05; **P < 0.01; ***P < 0.001; ****P < 0.0001 by 2-tailed *t* test (**B**, **D**, and **I**). NS, not significant. Scale bars: 100 μm (**C** and **F** [right]), 1 cm (**F**, left), and 50 μm (**I**).

To determine the role of GS in liver cancer, we used the sleeping beauty transposon and hydrodynamic tail vein injection (SB-HTVI) system to deliver c-Met and the constitutively active N-terminal deletion mutant of β-catenin (ΔN90-β-catenin), which has been characterized to induce HCC (23, 24). Similar levels and activity of ΔN90-β-catenin and c-Met were achieved in the WT and *Glul*-KO mice (Figure 1, A and B). As GS is a transcriptional target of β-catenin, GS expression was induced in the WT but not *Glul*-KO livers (Figure 1, A–C), whereas another β-catenin transcriptional target, Axin2, was induced to a similar extent in both mice (Figure 1B). Strikingly, *Glul*-KO mice displayed a significantly increased tumor burden (Figure 1D) and reduced survival, with a median survival of approximately 8.4 weeks in WT mice and 5.7 weeks in *Glul*-KO mice (Figure 1E). Livers harvested at approximately 6 weeks (endpoint of the *Glul*-KO mice) displayed solid-type HCC that was poorly differentiated in the *Glul*-KO mice, accompanied by increased levels of the proliferation marker PCNA and decreased expression of the UCEs ARG1 and CPS1 (Figure 1F). Therefore, genetic ablation of GS enhanced tumor development in the c-Met/β-catenin HCC mouse model.

To determine whether the increased tumor burden in *Glul*-KO livers was due to either early tumor initiation or progression, we injected oncogenes into *Glul^fl/fl^* mice first via SB-HTVI and then injected adenoviral GFP or adenoviral Cre via the tail vein to knock out GS expression 7 days after oncogene induction (Figure 1G). Like the Alb-Cre model, in which GS ablation occurred at the embryonic stage before oncogene activation, knocking out GS after oncogene expression also led to increased tumor burden (Figure 1H). We further examined the livers at various time points after c-Met/β-catenin SB-HTVI. The *Glul*-KO livers showed an accelerated progressive increase in dysplastic nodules, as observed by hematoxylin/eosin (H&E) staining and PCNA IHC (Figure 1I). These results, together with the observations that similar oncogenic activity was observed between the WT and *Glul*-KO livers (Figure 1, A and B), indicate that the increased tumor burden upon GS disruption was due to accelerated tumor progression. In the rest of our study, we mainly used liver samples harvested 2 to 3 weeks after oncogene injection, as the data suggest that this timing is sufficient for dysplastic tissue mass accumulation. This enabled the capture of molecular differences yet was early enough such that the changes were likely to be causative for tumor progression.

Importantly, in addition to the c-Met/β-catenin SB-HTVI model, we also tested several other models to determine whether the increased tumor progression in *Glul*-KO livers is a common phenomenon upon β-catenin hyperactivation. The chemical carcinogen diethylnitrosamine (DEN) has been shown to induce β-catenin mutations and HCC (25, 26). While DEN treatment led to a similar extent of DNA damage response and growth signaling in the WT and *Glul*-KO livers (Supplemental Figure 1F), *Glul*-KO mice displayed markedly increased tumor burden and cell proliferation upon treatment with DEN together with phenobarbital (PB) or a high-fat diet (HFD) (Supplemental Figure 1, G–J). Similarly, SB-HTVI of YAP/ΔN90-β-catenin also induced increased tumor burden in the *Glul*-KO liver (Supplemental Figure 1, K and L). Consistent with the activation of β-catenin, GS expression was induced in all these models. Interestingly, SB-HTVI of c-Met and single-guide RNA (sgRNA) of *Axin1* (sgAxin1), which has been shown to induce HCC but not GS expression (27), induced HCC to a similar extent in both WT and *Glul*-KO livers (Supplemental Figure 1, M and N). Taken together, these results strongly indicate that genetic disruption of GS leads to accelerated tumor progression in HCC driven by β-catenin and suggest that GS may have a tumor-suppressing function.

### GS ablation leads to increased mTORC1 signaling in response to β-catenin activation.

**Figure 2:**
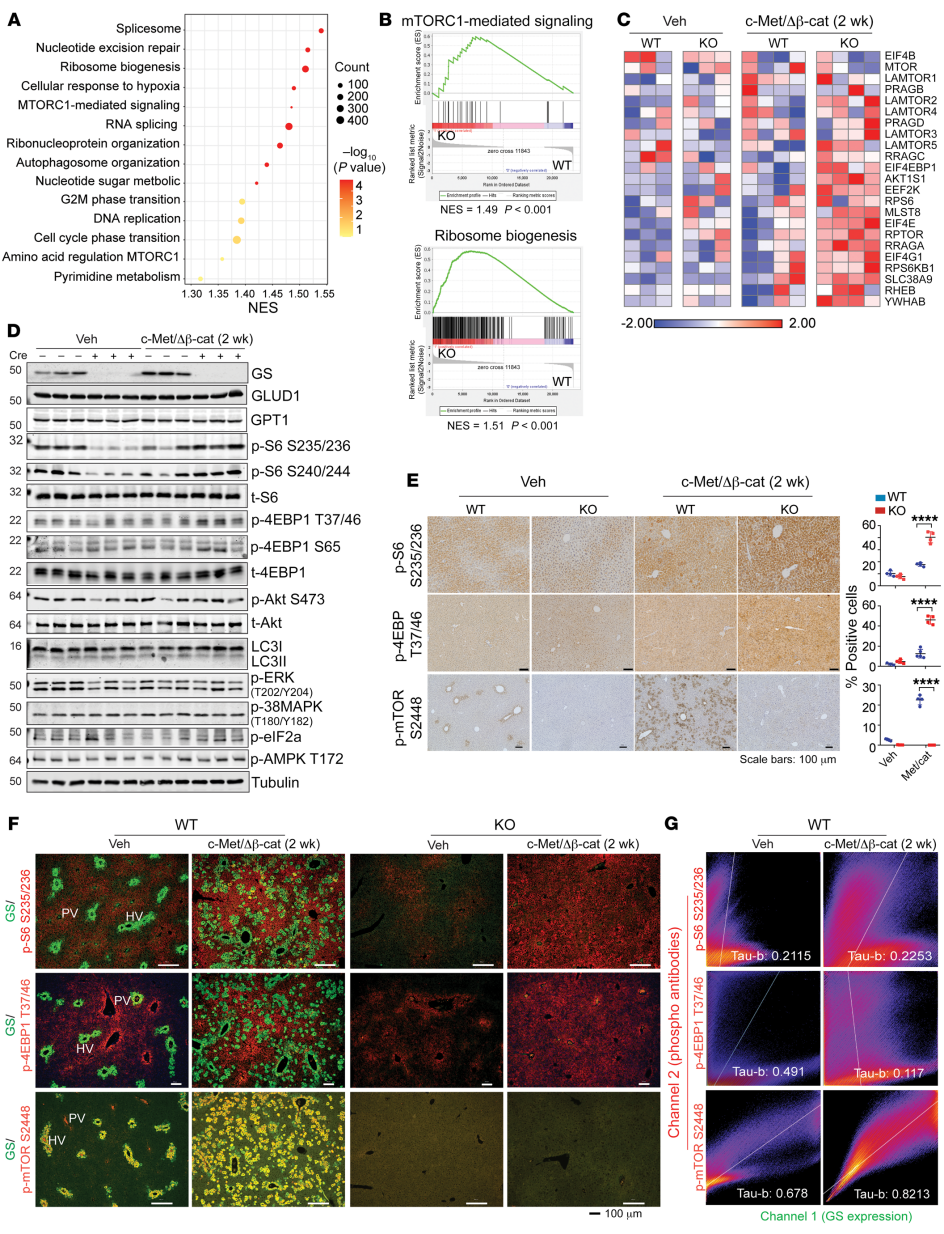
GS ablation leads to elevated mTORC1 activation in mouse livers harboring c-Met/ΔN90-β-catenin. Seven-week-old *Glul^fl/fl^* Alb-Cre^–^ (WT) and *Glul^fl/fl^* Alb-Cre^+^ (KO) male mice were injected with vehicle or c-Met/ΔN90-β-catenin/SB10 plasmids, and livers were harvested 2 weeks later. (**A**) RNA-seq was performed, and the summary of significantly changed pathways identified by GSEA (*n* = 4). NES, normalized enrichment score. (**B**) GSEA enrichment plots of mTORC1-mediated signaling (left panel) and ribosome biogenesis (right panel) that are positively enriched in KO livers (*n* = 4). (**C**) Heatmap of the relative expression of the indicated genes related to the mTORC1-mediated signaling pathway (*n* = 3–4). (**D**) Liver lysates were probed for the indicated proteins (*n* = 3). Lowercase “t” indicates that the total protein was probed. (**E**) IHC was performed (*n* = 3 mice for each group). The number of positive cells was quantified by ImageJ from 3–4 randomly selected fields. ****P < 0.0001 (2-tailed *t* test). (**F**) FFPE liver sections were costained for GS (green) and p-S6 S235/236 (red), p-4EBP1 T37/46 (red), or p-mTOR S2448 (red) (*n* = 3). The portal vein (PV) and hepatic vein (HV) were judged by GS expression in healthy WT livers. (**G**) Fluorescence images in **F** were subjected to colocalization analyses using the Coloc2 plugin in ImageJ. The pixel intensity correlation of channels 1 and 2 over space is depicted as a 2D scatterplot. Scale bars: 100 μm (**E** and **F**). Kendall’s Tau-b correlation analysis was used to test the significance of correlation and was judged positively correlated when greater than 0.5.

**Figure 3:**
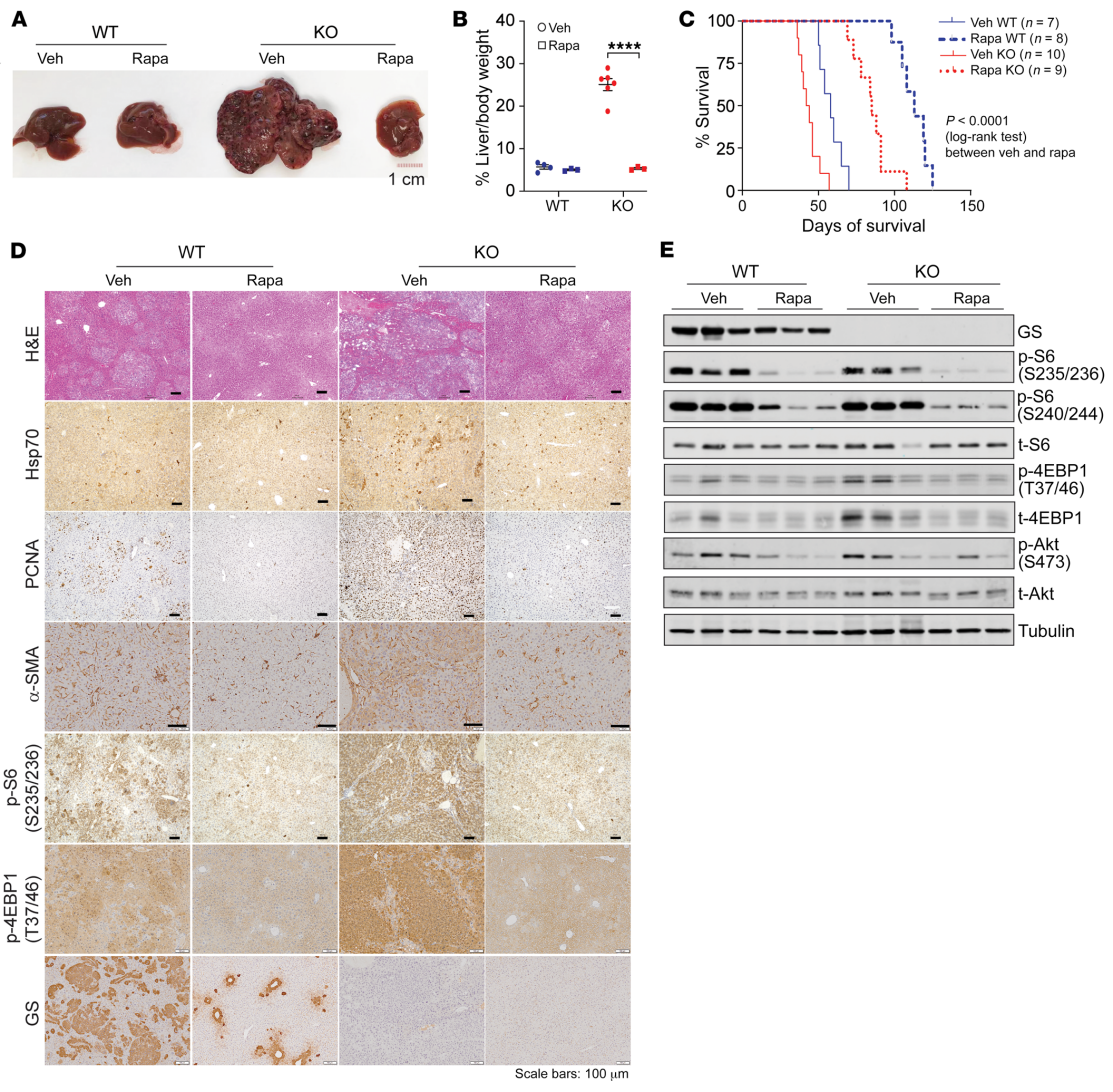
Rapamycin suppresses HCC development in GS-deficient livers. Seven-week-old *Glul^fl/fl^* Alb-Cre^–^ (WT) and *Glul^fl/fl^* Alb-Cre^+^ (KO) male mice were injected with c-Met/ΔN90-β-catenin/SB10 plasmids via SB-HTVI. On the same day, half of each group of mice were i.p. injected with rapamycin (Rapa), while the other half received vehicle (Veh; 0.4% DMSO in PBS; 3 times per week). (**A**) Livers were harvested 6 weeks after oncogene injection (*n* = 3–6). Scale bar: 1 cm. (**B**) Liver/body weight ratios were plotted and are shown as mean ± SEM (*n* = 6). ****P < 0.0001 (2-tailed *t* test). (**C**) Kaplan-Meier curves. (**D**) Liver tissue sections were stained with H&E and by IHC for the indicated proteins. Scale bar: 100 μm. (**E**) Liver lysates were probed for the indicated proteins.

We performed RNA sequencing (RNA-seq) analysis of liver tissues from WT or *Glul*-KO mice harvested 2 or 3 weeks after injection with c-Met/ΔN90-β-catenin (Supplemental Figure 2A). Differential gene expression analysis and gene set enrichment analysis (GSEA) were performed. Oncogene expression for 2 weeks in *Glul*-KO livers led to increased expression of genes enriched in pathways that included RNA splicing, ribosome biogenesis, nucleotide metabolism, and mTORC1 signaling (Figure 2, A and B), consistent with the accelerated tumor progression in the *Glul*-KO livers. Notably, the expression of a number of genes related to the mTORC1 signaling pathway was markedly higher in the *Glul*-KO livers than in the WT livers upon oncogene injection (Figure 2C), consistent with their accelerated growth. Immunoblotting (IB) showed that the basal phosphorylation levels of the direct mTORC1 substrate 4EBP1 (T37/46, S65) and a commonly used readout of mTORC1 activity, p-S6 (S235/236, S240/244), were lower in the *Glul*-KO livers than in the WT livers, indicating that mTORC1 signaling was lower in the KO livers (Figure 2D and Supplemental Figure 2B). However, c-Met/β-catenin led to a more marked increase in these phosphorylated molecules in the *Glul*-KO livers than in the WT livers at both 2 weeks (Figure 2D) and 3 weeks (Supplemental Figure 2B). Increased p-S6 and p-4EBP1 levels in oncogene-expressing *Glul*-KO livers were also observed by IHC (Figure 2E). In stark contrast, p-mTOR S2448, which has been suggested to not correlate with mTORC1 activity despite its frequent use as a marker for mTORC1 activation (28), was more substantially induced in the WT livers, and no p-mTOR S2448 signal could be detected in the *Glul*-KO livers (Figure 2E), indicating that p-mTOR S2448 is dependent on GS, consistent with a previous report (20). No apparent differences were observed in the levels of p-Akt (S473), p-ERK (T202/Y204), p-38 MAPK (T180/Y182), p-AMPK (T172), and LC3-II between the WT and *Glul*-KO livers at the 2- or 3-week time points after oncogene expression (Figure 2D and Supplemental Figure 2B), indicating that these signaling events and autophagy may not have a causative role or may be masked due to the small number of transforming cells at the early stage in accelerated tumor development in *Glul*-KO livers.

Our above data indicate that while GS is required for mTORC1 S2448 phosphorylation — which currently has unknown function — GS ablation can lead to increased mTORC1 activity upon c-Met/β-catenin activation, suggesting mTORC1 activation induced by c-Met/β-catenin may be independent of GS expression. To more closely examine the relationship between GS and mTORC1 activation in the liver, we performed immunofluorescence (IF). In the WT livers, c-Met/ΔN90-β-catenin induced midzonal expression of GS as expected (Figure 2F and Supplemental Figure 3A). p-mTOR S2448 colocalized with GS expression in the pericentral area in healthy livers and in the midzonal area upon oncogene activation (Figure 2, F and G, and Supplemental Figure 3B). In the *Glul*-KO livers, no p-mTOR S2448 signal could be detected (Figure 2F). However, p-S6 S235/236 and p-4EBP1 T37/46 were inversely correlated with GS expression in WT livers, i.e., GS^hi^ hepatocytes displayed decreased signals of these 2 phosphoproteins (Figure 2, F and G, and Supplemental Figure 3C). Consistent with our IB and IHC results (Figure 2, D and E, and Supplemental Figure 2B), oncogene activation led to increased p-S6 S235/236 and p-4EBP1 T37/46 in the *Glul*-KO livers (Figure 2F). Therefore, while GS expression is required for p-mTOR S2448, as previously reported (20), it appears to suppress mTORC1 activity, as indicated by p-S6 and p-4EBP1.

### Inhibition of mTORC1 suppresses HCC development.

As our above results show that GS ablation activates mTORC1 and promotes tumor progression, it became important to determine whether the accelerated tumor progression in the *Glul*-KO livers can be suppressed by mTORC1 inhibition. Markedly, rapamycin treatment led to a drastic reduction in tumor burden in *Glul*-KO livers (Figure 3, A and B) and prolonged animal survival (Figure 3C). IHC and IB analyses showed that rapamycin treatment led to decreased dysplasia (H&E staining and Hsp70 IHC), cell proliferation (PCNA), fibrotic response (α-SMA), and various mTOR signaling events (p-S6 235/236, p-S6 S240/244, p-4EBP1 T36/37, p-4EBP1 S65, p-Akt S473) (Figure 3, D and E). These results strongly indicate that mTORC1 activation plays a pivotal role in the accelerated onset of HCC driven by β-catenin in *Glul*-KO livers.

Rapamycin also led to increased survival and inhibition of tumor progression and mTOR signaling in the WT livers (Figure 3, C–E), indicating that mTOR activation also plays a tumor-promoting role in the GS-WT tumors, which is in agreement with a previous report (20). Interestingly, during tumor progression in WT livers, we noticed a progressive accumulation of GS^lo^ cells with high expression of β-catenin (Supplemental Figure 3A). These GS^lo^ tumor cell populations were high for p-4EBP1 yet low for p-mTOR S2448 (Supplemental Figure 3, B and C). These results suggest the possibility that (a) some β-catenin–transformed hepatocytes in the WT liver have become GS^lo^, which may have resulted in higher mTORC1 activity and a growth advantage over GS-positive cells, and (b) GS-positive tumor cells, albeit with decreased mTORC1 signaling, may still rely on mTORC1 for survival and proliferation.

### Oncogenic β-catenin activation leads to a decreased urea cycle.

**Figure 4:**
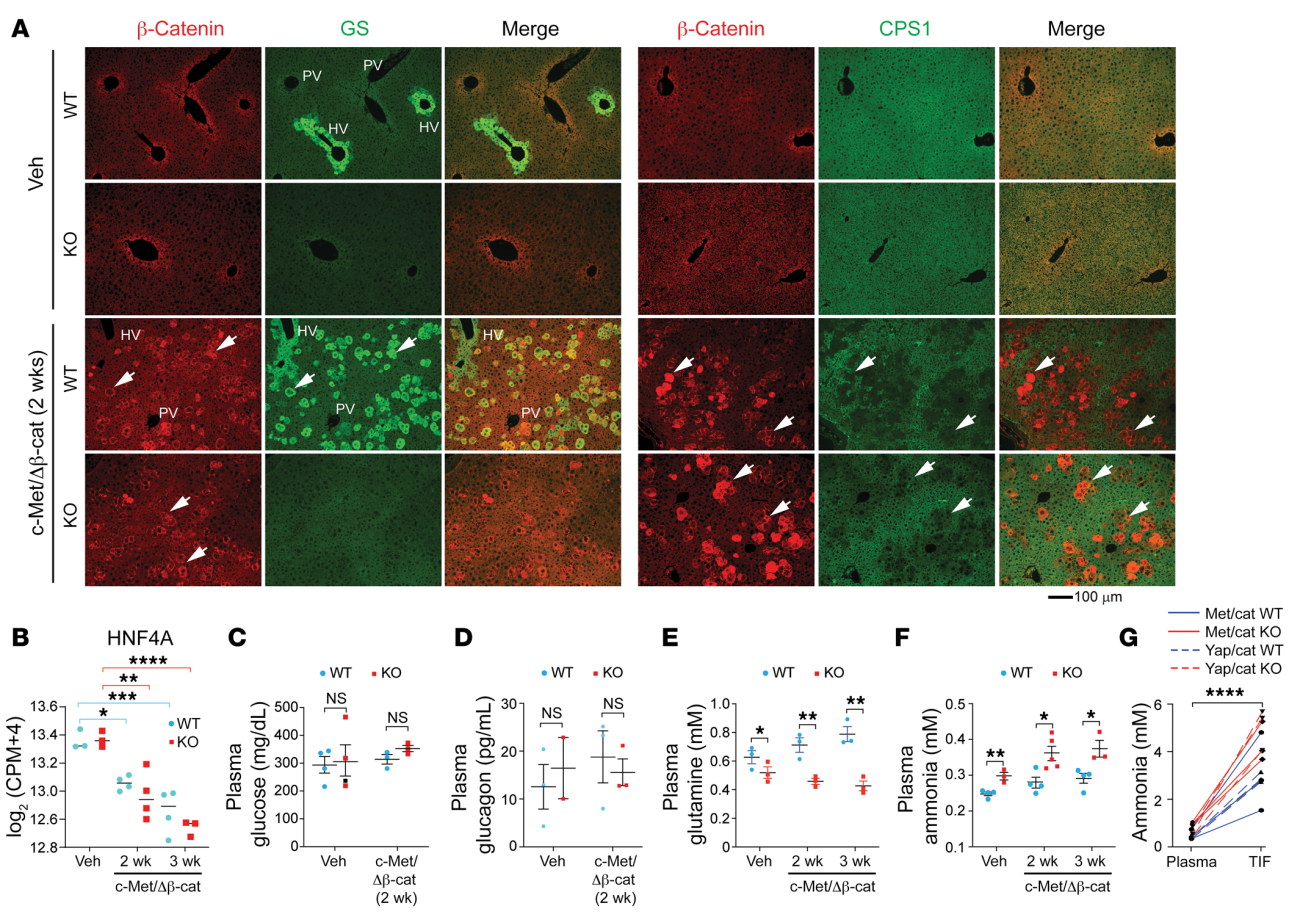
c-Met/ΔN90-β-catenin activation leads to decreased UCEs and defective ammonia handling. Seven-week-old *Glul^fl/fl^* Alb-Cre^–^ (WT) and *Glul^fl/fl^* Alb-Cre^+^ (KO) male mice were injected with vehicle or c-Met/ΔN90-β-catenin/SB10 plasmids via SB-HTVI. (**A**) Liver sections were harvested at 2 weeks and costained for β-catenin (red)/GS (green) or β-catenin (red)/CPS1 (green). The portal vein (PV) (negative for GS) and hepatic vein (HV) (positive for GS) are marked in the WT livers. Arrowheads point to a few cells that express β-catenin, which positively correlates with GS expression in WT livers and inversely correlates with CPS1 in both WT and KO livers. Scale bar: 100 μm. (**B**) RNA-seq analysis shows that the expression of the transcription factor HNF4A was suppressed in oncogene-expressing livers. (**C**–**F**) Plasma glucose (**C**), glucagon (**D**), glutamine (**E**), and ammonia (**F**) levels were determined for the indicated time points (*n* = 3–5). (**G**) Ammonia levels in the plasma and tumor interstitial fluid (TIF) were measured 6 weeks after c-Met/ΔN90-β-catenin injection (*n* = 3) and 9.6 weeks after YAP/ΔN90-β-catenin injection (*n* = 3), respectively. Each line represents 1 mouse, and linked ammonia results in plasma and TIF were obtained from the same mouse. The results are expressed as mean ± SEM. *P < 0.05, **P < 0.01, *8*P < 0.001, ****P < 0.0001 by 1-way ANOVA (**B**) or 2-tailed *t* test (**C**–**F**). NS, not significant.

A major metabolic function of GS in the liver of mammals is to assimilate and detoxify inorganic nitrogen waste. The homeostatic level of Wnt/β-catenin signaling plays a crucial role in liver development and function (29, 30). It is highly active in the pericentral area and low in the periportal and midzonal areas, which correlates positively with the expression of GS and inversely with UCEs, suggesting that β-catenin activation can lead to increased expression of GS and decreased expression of UCEs (11). Indeed, expression of c-Met/ΔN90-β-catenin induced GS expression in the WT liver and suppressed the expression of CPS1 (Figure 4A), ARG1 (Supplemental Figure 4A), ASS1 (Supplemental Figure 4B), and OTC (Supplemental Figure 4C) in both WT and *Glul*-KO livers. The suppression of the UCEs was likely due to the decreased expression of the transcription factor HNF4A (Figure 4B), which was previously suggested to drive the expression of the UCEs and can be suppressed by Wnt/β-catenin (31, 32). While oncogenic activation had no significant effect on the levels of plasma glucose and glucagon (Figure 4, C and D), it led to a greater difference in the level of plasma Gln (Figure 4E). Oncogenes also led to increased levels of ammonia in the plasma (Figure 4F) and in tumor interstitial fluid by both c-Met/ΔN90-β-catenin and YAP/ΔN90-β-catenin (Figure 4G), which were further increased in the *Glul*-KO mice (Figure 4, F and G). These results indicate that oncogenic β-catenin can suppress the expression of UCEs while activating the expression of GS. As suppression of UCEs leads to compromised ammonia clearance, upregulation of GS expression may help the liver cope with stress, and loss of GS exacerbates the hyperammonemia condition.

### GS ablation leads to increased production of nonessential amino acids.

**Figure 5:**
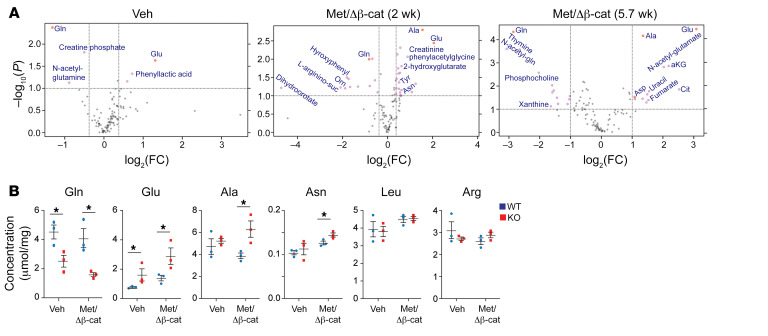
GS ablation enhances Glu-derived amino acids upon oncogene activation. Seven-week-old *Glul^fl/fl^* Alb-Cre^–^ (WT) and *Glul^fl/fl^* Alb-Cre^+^ (KO) male mice were injected with vehicle or c-Met/ΔN90-β-catenin/SB10 plasmids via SB-HTVI. Livers were harvested at the indicated time points. (**A**) Relative abundance of metabolites was determined by LC-MS. Volcano blots of differentially present metabolites between the KO and WT livers are shown. *x* axis: log_2_(fold change); *y* axis: –log_10_(*P* value). (**B**) Liver tissues were collected 2 weeks after oncogene injection. Absolute concentrations for the indicated amino acids were determined using spikes of respective standards. *P < 0.05 by 2-tailed *t* test in **B**.

We then performed nontargeted metabolomics analysis by liquid chromatography–coupled mass spectrometry (LC-MS) to compare metabolic differences between the *Glul*-KO and WT livers. The most significant changes were the decrease in Gln and increase in Glu in the *Glul*-KO livers compared with the WT livers (Figure 5A and Supplemental Figure 5, A and B), consistent with the GS function that condenses Glu and ammonia into Gln. This difference in the Glu/Gln cycle persisted during tumor progression (Figure 5A and Supplemental Figure 5, A–F). Notably, there was an increase in the pool size of Glu-derived nonessential amino acids (NEAAs), including Ala and Asp, with Ala being more profoundly elevated (Figure 5A and Supplemental Figure 5, A–F). The drastic decrease in Gln and reciprocal increase in Glu and Ala in the *Glul*-KO livers upon oncogene activation were also determined by measuring their absolute concentrations in the liver (Figure 5B). As the tumors progressed to later stages, other growth/proliferation-related metabolites, including Arg and pyrimidines, also increased in the *Glul*-KO livers, as indicated by the respective pool sizes and increased phosphorylation of CAD (carbamoyl-phosphate synthetase 2, aspartate transcarbamylase, and dihydroorotase) (Supplemental Figure 5, E–G).

**Figure 6:**
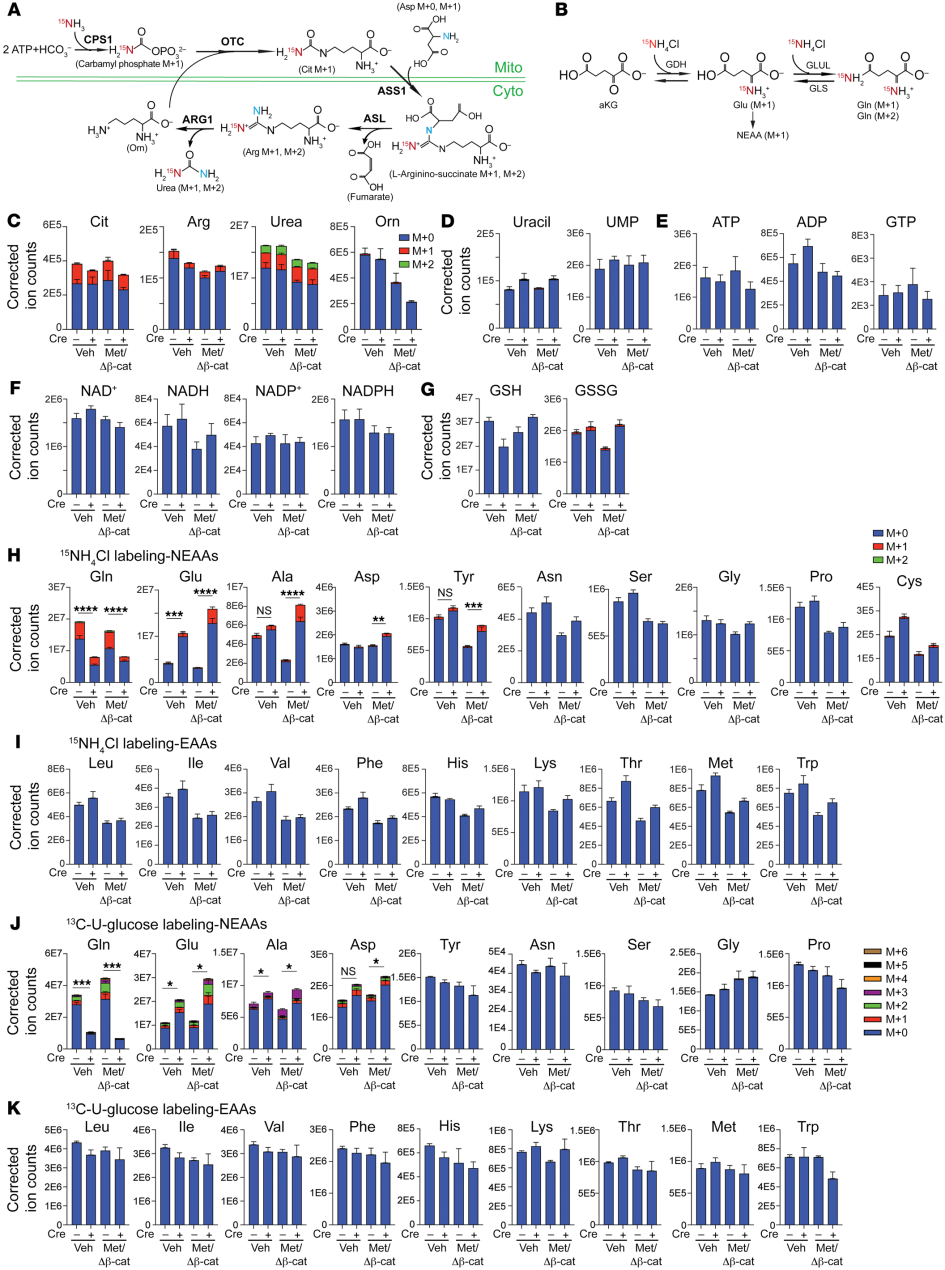
GS ablation leads to decreased Gln and increased Glu and Glu-derived NEAAs. (**A**) Schematic illustration of ^15^N-NH_4_Cl metabolic fates via the urea cycle. (**B**) Schematic illustration of ammonia nitrogen assimilation via Glu and Gln biosynthesis. (**C**–**I**) Two weeks after oncogene injection, mice were i.p. injected with 5 mmol/kg ^15^N-NH_4_Cl. Liver tissues were collected and snap-frozen after 30 minutes. Total labeled and unlabeled metabolite ion counts for urea cycle–related metabolites (**C**), metabolites related to pyrimidine synthesis (**D**), bioenergy (**E**), redox (**F**), oxidative stress (**G**), nonessential amino acids (NEAAs) (**H**), and essential amino acids (EAAs) (**I**), are shown. (**J** and **K**) Mice injected with oncogenes for 2 weeks were i.p. injected with 5 mmol/kg ^13^C-U-glucose. Liver tissues were collected 30 minutes later. Labeled fractions of NEAAs (**J**) and EAAs (**K**) are shown as mean ± SEM (*n* = 3–6 in each group). *P < 0.05; **P < 0.01; ***P < 0.001; ****P < 0.0001 by 2-tailed *t* test (**H** and **J**). NS, not significant.

To further delineate the metabolic changes upon GS ablation and oncogene activation, we performed stable isotope tracing using ^15^N-labeled ammonium (^15^N-NH_4_Cl for i.p. bolus and ^15^N-NH_4_OAc for jugular vein infusion) to examine the 3 metabolic fates of ammonia in the liver: (a) citrulline (Cit) as an index of abundance in the carbamoyl phosphate pool and urea cycle (Figure 6A), (b) Glu as an index of GDH activity, and (c) Gln as an index of GS activity (Figure 6B) (33). We first compared the labeling efficiency of i.p. bolus injections for 30 minutes or 4 hours and an i.v. infusion via the jugular vein for 2.5 hours using ^15^N-NH_4_^+^ in healthy WT mice. Boluses administered i.p. for 30 minutes led to a markedly higher enrichment of labeled Cit, Glu, and Gln, which were diminished at the 4-hour time point (Supplemental Figure 5H), consistent with the rapid turnover of ammonia in the liver, as previously reported (34). ^15^N-NH_4_^+^ i.v. 2.5-hour infusion showed that the circulating ammonium precursor was enriched consistently among the 4 groups of mice, and that the labeling efficiency of the main Glu-Gln cycle metabolites was similar to that in the 30-minute i.p. bolus injection (Supplemental Figure 5I). On the other hand, while the 30-minute i.p. bolus reached an enrichment of Cit, Glu, and Gln similar to that of the 2.5-hour i.v. infusion, the increased Glu labeling and decreased Gln labeling in the *Glul*-KO livers were more evident in the 30-minute i.p. bolus labeling (Supplemental Figure 5J), likely due to substrate recycling in the 2.5-hour infusion procedure. Moreover, since peritoneal absorption mainly occurs through the portal venous system and bypasses other organs, it is believed to reflect liver metabolism more faithfully. We therefore mainly used 30-minute i.p. boluses to assess the direct metabolic fate of ammonia in the liver. It is also important to note that the *Glul*-KO mice were more susceptible to ammonia toxicity; hence, we used a relatively low dose of ^15^N-labeled ammonia (5 mmol/kg) as the tracer.

We then compared the metabolic fates of ^15^N-labeled ammonia in *Glul*-KO and WT mice at the basal state or upon 2-week oncogene activation. We observed strong enrichment of Cit, Arg, and urea and no labeling of ornithine (Orn), as expected (Figure 6C). Both the pool sizes of urea and Orn were markedly reduced upon oncogene activation (Figure 6C), consistent with the decreased expression of ARG1, which cleaves Arg into urea and Orn (Supplemental Figure 4). The relatively unchanged pools of Cit and Arg were likely to be the result of blockade of the urea cycle flux due to decreased expression of other UCEs. Taken together, these results show that c-Met/β-catenin activation led to decreased UCE expression (Figure 4A and Supplemental Figure 4), increased plasma and interstitial ammonia levels (Figure 4, D and E), and decreased urea cycle output (Figure 6C), all of which were exacerbated in *Glul*-KO livers. There was little to no labeling of uracil and UMP (Figure 6D), bioenergy (Figure 6E), NAD+/NADH, and NADP+/NADPH (Figure 6F), and the relative abundance of these molecules (*m*+0) was similar between the *Glul*-KO and WT livers with 2 weeks of oncogene activation. Increased glutathione disulfide (GSSG) levels were detected in the *Glul*-KO livers, suggesting increased oxidation (Figure 6G).

For the Glu/Gln ammonia assimilation routes, there was a strong enrichment of ^15^N-Gln in the WT livers, with 98.4% labeled fraction being *m*+1 and 1.6% being *m*+2. The *m*+1 Gln was determined to be predominantly labeled at the terminal amide group (Supplemental Figure 5K). The ratios between Gln (*m*+1)/Glu (*m*+0) and Gln (*m*+2)/Glu (*m*+1) were similar in the WT livers under all conditions (Supplemental Figure 5L). These data indicate that the labeled Gln was from GS activity. Indeed, both the enrichment and pool size of labeled Gln were drastically reduced in the *Glul*-KO livers, which was further exacerbated upon oncogene activation (Figure 6H).

Reciprocally, the pool sizes of Glu and its labeled fraction were significantly higher in the *Glul*-KO livers, which were further increased upon oncogene activation (Figure 6H). Of the NEAAs that can be derived from Glu, we observed ^15^N-labeled Ala, Tyr, and Asp, of which Ala had the highest enrichment ratio (Figure 6H). In contrast, there was little to no ^15^N labeling of other nitrogen-containing molecules, including essential amino acids (EAAs) (Figure 6I). Therefore, the most drastic metabolic differences between the *Glul*-KO and WT livers with oncogene activation were Glu and Glu-derived Ala, as also indicated by measuring the absolute concentrations of several amino acids (Figure 5B).

Oncogenic transformation is known to promote glucose metabolism to support increased bioenergetic and biosynthetic needs. For the aKG-Glu-Gln nitrogen assimilation pathway and NEAA production, glucose metabolism also plays an important role in providing α-ketoacids as nitrogen acceptors. We therefore examined the metabolic fate of glucose using ^13^C-U-glucose as the tracer. Oncogene activation led to an overall increase in the labeling of glycolytic and TCA cycle intermediates in both the liver and plasma (Supplemental Figure 5M). While most of the glycolysis and TCA metabolites did not show a drastic difference between the WT and *Glul*-KO livers, there was a marked decrease in the enrichment of labeled Gln and a reciprocal increase in Glu in *Glul*-KO livers with oncogene activation (Supplemental Figure 5M). Notably, a higher enrichment of *m*+3 ^13^C-Ala, which indicates pyruvate as the carbon source for Ala, was observed in the oncogene-expressing *Glul*-KO livers (Figure 6J). Labeled Asp was also observed, whereas the other NEAAs and EAAs were not labeled (Figure 6, J and K). Taken together, these results indicate that β-catenin activation leads to a decreased urea cycle and increased ammonia burden. A disruption of the Gln ammonia assimilation can cause metabolic alterations, including drastic accumulation of Glu and Glu-derived Ala and Asp.

### Glu-derived NEAAs activate mTORC1.

**Figure 7:**
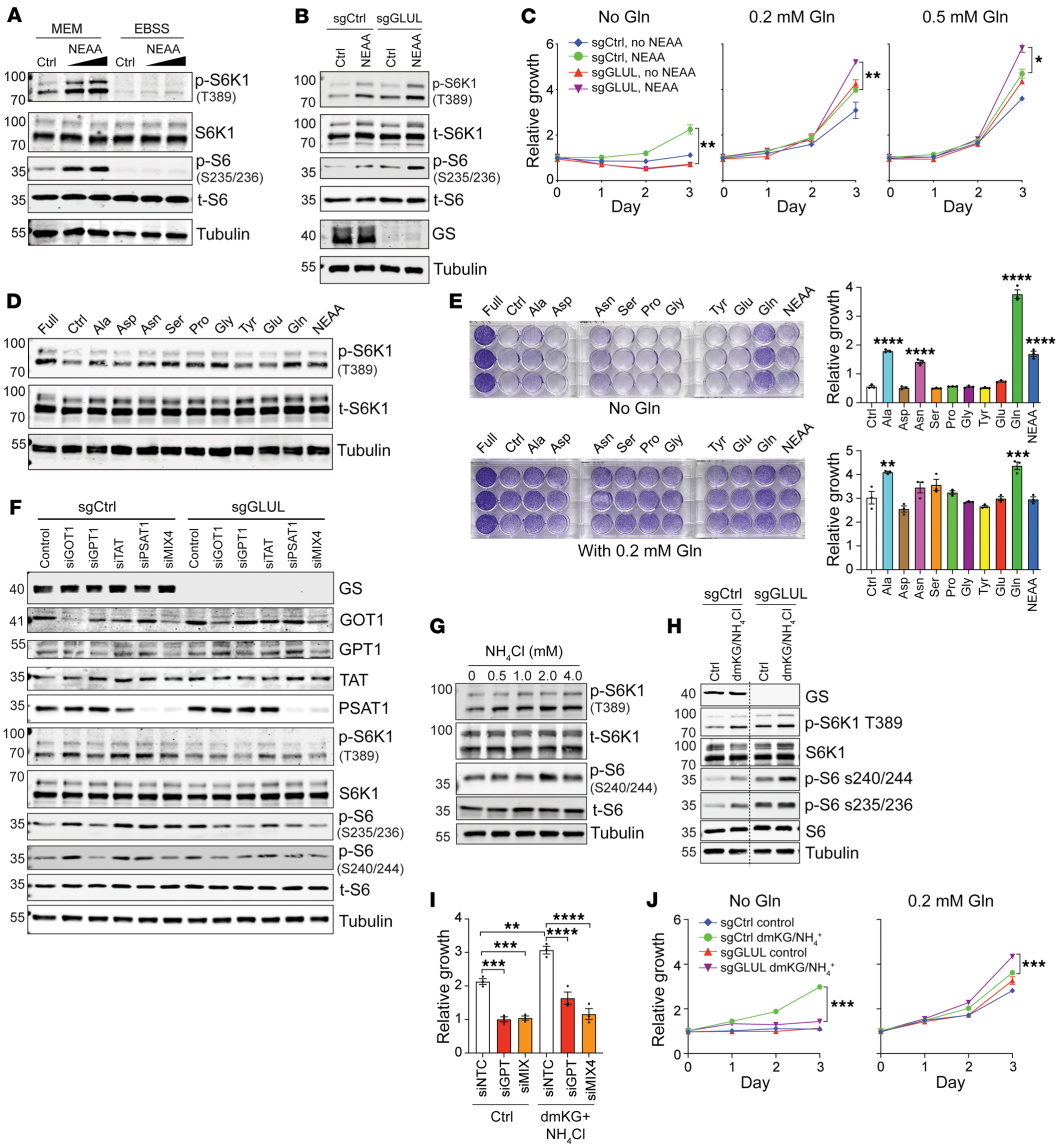
Glu-derived Ala stimulates mTORC1 and promotes Hep3B cell proliferation. (**A**) Hep3B cells prestarved in MEM or EBSS for 8 hours were left unstimulated (Ctrl) or stimulated for 30 minutes with 1× or 2× NEAA mix. Immunoblotting was performed. (**B**) Cells stably expressing sgCtrl and sg*GLUL* were prestarved in MEM followed by 30-minute treatment with 1× NEAA mix. Immunoblotting was performed. (**C**) sgCtrl and sg*GLUL* cells in various amounts of Gln supplied with 0 or 1× NEAA mix. Relative cell growth is shown. (**D**) Cells prestarved in MEM for 4 hours were left unstimulated (Ctrl) or stimulated for 30 minutes with 1 mM indicated amino acids or a 1× NEAA mix. Cells cultured in full medium (MEM + 10% FBS + 1× NEAA mix) were used as a positive control. (**E**) Cells were cultured in 0 or 0.2 mM Gln supplied with individual NEAAs or a 1× NEAA mix. Relative growth compared with the control is shown. (**F**) sgCtrl and sg*GLUL* cells were transfected with indicated individual siRNAs or a mix of all 4 siRNAs (siMIX4) for 48 hours. Cells were cultured in MEM for 9 hours and harvested. (**G** and **H**) Indicated cells were prestarved in MEM for 8 hours, and then were treated with NH_4_Cl for 24 hours (**G**) or with 1.5 mM dmKG plus 4 mM NH_4_Cl for 1 hour (**H**). (**I**) Cells transfected with siControl, si*GPT1*, or siMIX4 for 48 hours were cultured in MEM containing 0.2 mM Gln without or with dmKG plus NH_4_Cl for 72 hours. Relative cell growth is shown (*n* = 3). (**J**) sgCtrl and sg*GLUL* cells were cultured in MEM containing 0 or 0.2 mM Gln and supplied with dmKG plus NH_4_Cl. Relative growth is shown (*n* = 3). *P < 0.05; **P < 0.01; ***P < 0.001; ****P < 0.0001 by 2-tailed *t* test (**C** and **J**) or 1-way ANOVA (**E** and **I**).

We next attempted to determine whether the metabolic alterations can affect mTORC1 activity. While much attention has been focused on mTORC1 activation by Leu, Arg, Met, and Gln (35–38), other amino acids, including Ala, have also been reported to activate mTORC1 (39–41). We then went on to determine whether Glu-derived NEAAs can stimulate mTORC1. We cultured multiple cancer cell lines in minimum essential media (MEM), which contains EAAs, or in Earle’s balanced salt solution (EBSS), an isotonic buffer solution that contains inorganic salts and glucose, both containing dialyzed fetal bovine serum (FBS). A NEAA mixture solution was added to stimulate the cells, and mTORC1 activation was assessed by p-S6K (T389), p-S6 (S235/236), or p-S6 (S240/244). Despite a few occasions where the phospho-antibodies did not show consistent results among the cell lines, NEAAs generally induced mTORC1 activation in a dose-dependent manner in Hep3B cells (Figure 7A) and in multiple other cell lines (Supplemental Figure 6, A–H) cultured in MEM. It is important to note that this NEAA-induced mTORC1 activation was diminished in EBSS, indicating that EAAs are prerequisites for NEAA-induced mTORC1 activation (Figure 7A and Supplemental Figure 6, A–E). Interestingly, both basal and NEAA-induced mTORC1 activation were further enhanced when GS was silenced in Hep3B and HepG2, two liver cancer cell lines that express a considerable amount of endogenous GS (Figure 7B and Supplemental Figure 6I). Consistent with previous reports that GS is important for nitrogen anabolism and cell growth by synthesizing Gln from other nitrogen sources under Gln starvation (15, 17, 18), in basal media that did not contain Gln, NEAAs could stimulate cell growth, which was abolished when GS was silenced (Figure 7C). However, when Gln was supplied in the culture media, silencing of GS led to more rapid growth (Figure 7C), consistent with a model that when the minimum requirement for Gln is met, GS ablation can enhance mTORC1 activation and cell growth.

We then added individual amino acids to MEM (containing EAAs) to determine which NEAAs can activate mTORC1 and stimulate cell growth. In various cell lines, Ala, Asn, Ser, Pro, and Gly stimulated mTORC1, as did Gln and the NEAA mix (Figure 7D and Supplemental Figure 6, J and K). Among these amino acids, Gln was clearly important for cell growth, and Ala and Asn could also enhance cell growth, even in the absence of Gln (Figure 7E). These results, together with our in vivo results that GS ablation leads to increased nitrogen assimilation into Ala (Figure 5C), indicate that increased hepatic Ala may facilitate mTORC1 activation and HCC development when GS is disrupted. Ala can be produced by the reversible glutamic-pyruvic transaminase (GPT), also called alanine aminotransferase (ALT), which catalyzes the transamination from Glu and pyruvate to Ala and aKG. There are 2 isoforms of GPT; GPT1 is cytosolic and predominantly expressed in the liver, intestines, and kidney, whereas GPT2 is mitochondrial and primarily expressed in the heart, pancreas, and brain (42). We therefore knocked down GPT1, as well as several other Glu aminotransferases, glutamic oxaloacetic transaminase 1 (GOT1), tyrosine aminotransferase (TAT), and phosphoserine transaminase 1 (PSAT1), to test their effects on mTORC1 activation and cell growth. While si*TAT* did not achieve successful silencing of TAT, the other siRNAs and the mix of all 4 siRNAs led to a significant decrease in the respective transaminase, among which si*GPT1* and siMix4 led to the most significant decrease in mTORC1 in both sgControl and sg*GLUL* cells (Figure 7F). These results indicate an mTORC1-stimulating role of Ala that is derived from Glu, in agreement with a previous finding that mTORC1 activation stimulated by Gln supplementation is dependent on Gln’s conversion into NEAAs via glutaminolysis and can be suppressed by the inhibition of glutamic aminotransferases (41).

It has been previously shown that GDH-mediated Glu synthesis from ammonia can stimulate amino acid production and cell growth (19). Indeed, ammonia alone or together with the cell-permeant dimethyl-ketoglutarate (dmKG) stimulated mTORC1 activation, as indicated by p-S6K1 and p-S6 (Figure 7, G and H), which was further enhanced upon GS silencing with exogenous Gln supplementation (Figure 7H). Similar to NEAAs, ammonia/dmKG stimulated cell growth that was diminished by si*GPT1* and siMix (Figure 7I). The cell growth stimulated by ammonia/dmKG was abolished by GS silencing in Gln-free media but enhanced in Gln-replete media (Figure 7J). Taken together, our above data indicate that Glu-derived NEAAs, especially Ala, can stimulate mTORC1 activation and cell growth, and this process is enhanced by GS ablation.

### Inhibition of GPT suppresses HCC growth in GS-deficient livers.

**Figure 8:**
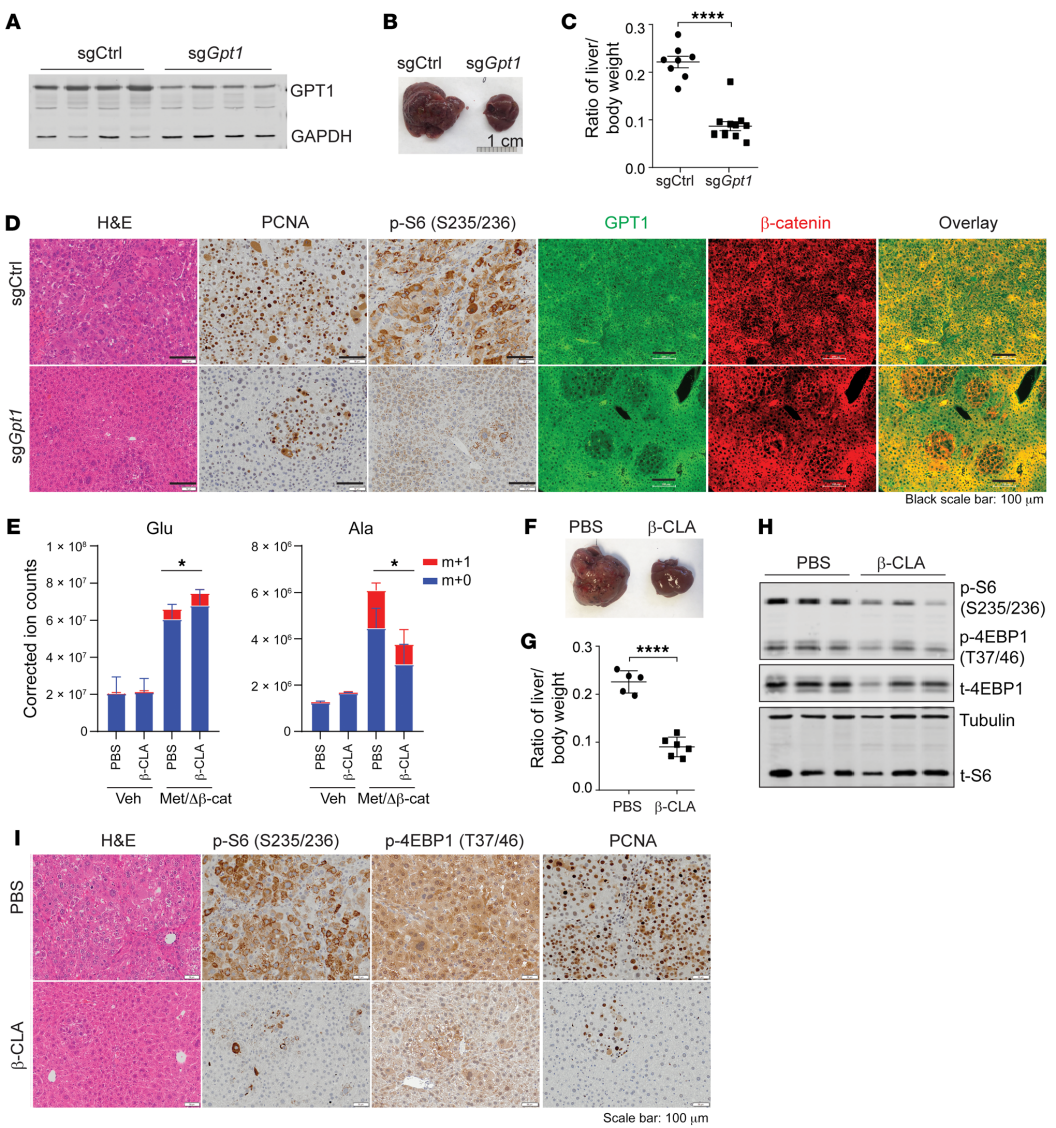
Inhibition of GPT1 suppresses HCC development in *Glul*-KO livers. (**A**–**D**) *Glul*-KO mice were injected with c-Met/ΔN90-β-catenin, together with sgCtrl (*n* = 8) or sg*Gpt1* (*n* = 10) plasmids, via SB-HTVI. Mice were sacrificed 6 weeks later (endpoint of the sgCtrl mice). (**A**) Successful GPT1 knockdown. (**B**) Representative gross liver images (*n* = 8–10). (**C**) Liver/body weight ratios (*n* = 8–10). (**D**) Liver tissues were stained with H&E and by IHC and IF. Note that while sgCtrl mice developed full-blown tumors that were positive for both GPT1 and β-catenin, sg*Gpt1* mice showed smaller tumor nodules with reduced GPT1 expression and low p-S6 (S235/236) despite high β-catenin expression. Scale bars: 50 μm (left panels), 100 μm (right panels). (**E**–**I**) *Glul*-KO male mice were injected with c-Met/ΔN90-β-catenin/SB10. On the same day, half of each group of mice were i.p. injected with β-CLA (20 mg/kg; 3 times per week), while the other half received PBS. (**E**) Two weeks later, mice were i.p. injected with 5 mmol/kg ^15^N-NH_4_Cl and sacrificed after 30 minutes. Total labeled and unlabeled metabolite ion counts for Glu and Ala were determined by LC-MS. (**F**–**I**) Mice were sacrificed 6 weeks later (endpoint of PBS-treated mice). (**F**) Representative gross liver images. (**G**) Liver/body weight ratios (*n* = 5 or 6). (**H**) Immunoblotting of liver lysates. (**I**) Liver tissue H&E and IHC. Scale bars: 50 μm. *P < 0.05; ****P < 0.0001 by 2-tailed *t* test (**C**, **E**, and **G**).

We then went on to test whether inhibition of Glu-derived NEAA production could suppress HCC development in vivo. Several glutamine metabolic enzymes, including GLUD1, GPT1, GLS1, and GLS2, did not show altered expression levels in *Glul*-KO livers or upon oncogene activation (Figure 2D and Supplemental Figure 6L). To test whether inhibition of GPT can suppress β-catenin–driven HCC development that was accelerated by loss of GS, GPT1 was successfully silenced by an sgRNA (Figure 8A), which significantly reduced the tumor burden in *Glul*-KO livers (Figure 8, B and C). While sgCtrl mice developed full-blown tumors, mice expressing sg*Gpt1* showed smaller tumor nodules with reduced GPT1 expression, as well as reduced PCNA and S6 phosphorylation despite Δ90-β-catenin expression (Figure 8D). Moreover, 2 permissive GPT inhibitors D,L-cycloserine (DL-Cyc) and β-chloro-L-alanine (β-CLA) markedly reduced the intracellular levels of Ala and cell growth (Supplemental Figure 6, M and N). Strikingly, in vivo administration of β-CLA, while reducing the level of Ala to a similar extent as in vitro (Figure 8E), largely reduced the tumor burden (Figure 8, F and G) that was accompanied by reduced mTORC1 and cell proliferation indicated by p-S6, p-4EBP1, and PCNA (Figure 8, H and I). These data strongly indicate that inhibition of glutamate transaminases is a viable strategy for treating HCCs that involve dysregulated ammonia clearance.

### Defective nitrogen waste removal in HCC driven by β-catenin mutations.

**Figure 9:**
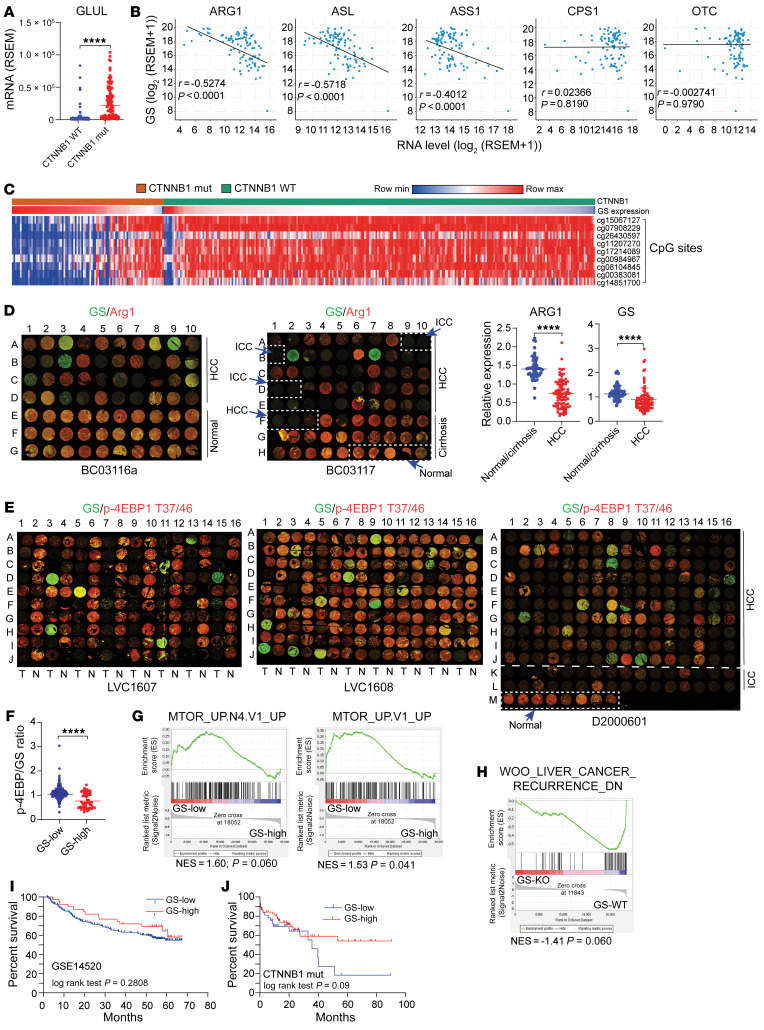
Defective nitrogen waste removal in HCC clinical samples. (**A**) Relative *GLUL* mRNA levels in liver cancer patients with WT or mutated *CTNNB1* (TCGA) were compared and are expressed as mean ± SEM. (**B**) Correlations between hepatic *GLUL* and UCE expression in patients with *CTNNB1* mutations (TCGA) was calculated by Pearson’s correlation. (**C**) *CTNNB1* mutation correlates positively with hepatic GS expression but inversely with *GLUL* CpG island methylation. (**D**) Two liver TMAs were costained for GS (green) and ARG1 (red) and quantified by ImageJ and normalized to the respective median (right panel). (**E**) TMAs with HCC tumors (T), adjacent normal tissue (N), or intrahepatic cholangiocarcinoma (ICC) were costained for GS (green) and p-4EBP1 T37/46 (red). (**F**) Ratios of p-4EBP1 T37/46 to GS intensities were plotted. Results are expressed as mean ± SEM. The cutoff value for GS-high (*n* = 52) versus -low (*n* = 372) was determined using the best separation between the 2 modes of a bimodal distribution. ****P < 0.0001 by 2-tailed *t* test (**A**, **D**, and **F**). (**G**) GSEA enrichment plots show that mTORC1 target genes correlate inversely with GS level in *CTNNB1*-mutated patients (TCGA). The cutoff value for GS-high (*n* = 62) versus -low (*n* = 34) was determined using the best separation between the 2 modes of a bimodal distribution. *P* value was calculated by estimated score in GSEA. (**H**) The TCGA data of the indicated study were stratified into the RNA-seq results obtained from WT and *Glul*-KO mice 2 weeks after c-Met/ΔN90-β-catenin injection (*n* = 4 in each group). GSEA plots show that high GS expression correlates with low recurrence. (**I**) Kaplan-Meier survival curves of patients with high (*n* = 41) versus low (*n* = 201) hepatic GS expression (GSE14520; *P* = 0.28, log rank). (**J**) In HCC patients harboring *CTNNB1* mutations (TCGA) as shown in **G**, high GS expression tends to associate with better survival (*P* = 0.09, log rank).

In HCC, UCEs are predominantly downregulated (43, 44), whereas GS is generally upregulated. Indeed, analysis using The Cancer Genome Atlas (TCGA) showed a strong correlation between GS expression and mutations in *CTNNB1*, the gene that encodes β-catenin (Figure 9A). In *CTNNB1*-mutated patients, there was an inverse correlation between GS expression and that of ARG1, ASL, and ASS1 (Figure 9B). Interestingly, in patients with *CTNNB1* mutations, GS expression strongly correlated with the methylation status of CpG islands, where GS expression was low in patients with hypermethylated *GLUL* (Figure 9C). Examination of clinical samples in tissue microarrays (TMAs) that contained 88 cases of HCC and 58 cases of normal/cirrhosis samples showed significantly lower expression of ARG1 in HCC (Figure 9D). The expression of GS, while drastically higher in some HCC cases, was markedly lower in others (Figure 9D). Importantly, using 2 TMA sets, each containing 80 HCC and their adjacent normal tissues (LVC1607 and LVC1608), and 1 TMA set that contained 160 HCC, 32 ICC, and 8 normal cases (D2000601), IF analysis showed that while GS and p-4EBP1 (T37/46) were more uniformly expressed in the normal tissues, their expression varied drastically in the HCC samples (Figure 9E). In these HCC tissues, whose *CTNNB1* status was unavailable, higher GS expression did not correlate with a higher p-4EBP1 level (Figure 9F). Using mTORC1-associated gene lists from 2 independent studies (45, 46), GSEA enrichment plots showed that GS expression correlated inversely with mTORC1 gene targets in *CTNNB1*-mutated cases (Figure 9G). Moreover, the TCGA data of a previous study (47) were stratified into the RNA-seq results obtained from WT and *Glul*-KO mice 2 weeks after c-Met/ΔN90-β-catenin injection, and GSEA showed that high GS expression was associated with low recurrence (Figure 9H). Finally, GS-low HCC patients showed an overall trend of poorer survival (Figure 9I), which was more significant in *CTNNB1*-mutated patients (Figure 9J). Taken together, these results indicate that while HCC patients, especially those with *CTNNB1* mutations, have an overall defective urea cycle and increased expression of GS, there exists a group of patients with low GS expression. The failure of both ammonia-handling mechanisms may contribute to HCC malignancy by enhancing mTORC1 signaling.

## Discussion

Ammonia is toxic to all vertebrate animals. In mammals, ammonia waste is largely produced by gastrointestinal bacteria and is detoxified via the urea cycle and Gln synthesis. Increased expression of urea cycle genes has been implicated in tumor promotion in various tumor types via mechanisms such as enhanced pyrimidine and polyamine production (14, 48, 49). However, in the liver, a defective urea cycle has been found to be tumor promoting (13, 50–52). Urea cycle deficiency can result from genetic mutations and is associated with liver cirrhosis (53, 54). Here, we found that oncogenic β-catenin activation leads to decreased expression of UCEs (Figure 4 and Supplemental Figure 4). Importantly, while β-catenin activation drives GS expression, we show here that genetic ablation of GS promotes HCC development. Therefore, our findings uncover a scenario in which β-catenin activation can lead to decreased UCE expression, which plays a tumor-promoting role, and increased GS expression, which plays a tumor-suppressing role. While it is seemingly paradoxical that β-catenin activation would lead to simultaneous decreased UCE expression and increased GS expression, it is not entirely surprising, as it appears to be a function of even the physiological level of Wnt/β-catenin signaling. The Wnt/β-catenin pathway plays an essential role in liver zonation patterning, which positively correlates with high GS expression in the pericentral region and inversely correlates with high UCE expression in the periportal and midzonal zones. Our current findings indicate the importance of maintaining hepatic nitrogen homeostasis that involves both the urea cycle and Glu/Gln ammonia detoxification pathways. As oncogenic β-catenin suppresses UCE expression, GS is upregulated for hepatocytes to cope with the compromised ammonia handling capacity. Cells that find a way to suppress GS expression gain growth advantages during oncogenic transformation. Indeed, even in HCC patients with β-catenin mutations, despite the general upregulation of GS expression, GS expression was found to be low in some cases that are associated with *GLUL* CpG island methylation (Figure 9C).

Gln anabolism, which is carried out by GS, has been recognized to promote oncogenesis and therapy resistance by producing Gln as a nitrogen donor for nitrogen-containing macromolecules such as nucleotides and hexosamines (15, 17, 18, 21, 55, 56). This is particularly important in poorly vascularized tumors such as PDAC, where the circulatory supply of Gln is limited (15). Here, we show that, unlike in many tissues, genetic ablation of GS in the liver leads to enhanced HCC development in several mouse models where β-catenin is activated and GS expression induced (Figure 1 and Supplemental Figure 1). Accelerated HCC development is associated with compromised nitrogen waste clearance, increased production of Glu-derived NEAAs, and subsequent mTORC1 activation.

Unlike other tissues, the liver receives portal blood that contains a significant amount of Gln, even in *Glul*-KO mice (Figure 5B) (8, 9). While this amount of Gln may be sufficient to support the nitrogen anabolic needs of hepatocytes, our findings suggest a previously unappreciated model in which the nitrogen waste clearance function of GS plays a more predominant role than its Gln-generation function to maintain liver homeostasis, especially under β-catenin activation. GS may suppress HCC development by reducing the hepatic levels of ammonia-, Glu-, and Glu-derived NEAAs. It is interesting to note that unlike in mammals where ammonia waste is converted to urea and Gln, in fish, ammonia is directly excreted through the gill and skin; hence, the Gln-producing function of GS is predominant in the liver, which may explain the protumorigenic role of GS in a YAP-driven zebrafish liver cancer model (21).

We report here that accelerated HCC development upon GS ablation is dependent on increased mTORC1 activation. The increased mTORC1 signaling in the *Glul*-KO liver upon oncogene activation was judged by RNA-seq (Figure 2, A–C, and Supplemental Figure 2A) and IB, IHC, and IF using several established mTORC1 markers, including p-S6 (S235/236), p-S6 (S240/244), p-4EBP1 (T37/46), and p4EBP1 (S65) (Figure 2, D–F, and Supplemental Figure 2, B and C). Moreover, rapamycin treatment largely repressed tumor progression even in the *Glul*-KO livers (Figure 3C). On the other hand, GS expression was inversely correlated with p-S6 and p-4EBP1 in both healthy and oncogene-expressing WT livers, consistent with the model that GS expression suppresses mTORC1 activation and tumorigenesis. Significantly, p-mTOR S2448, which has been frequently used as an activation marker for mTORC1, correlated with GS expression in both healthy and oncogene-expressing WT livers but was not detected in *Glul*-KO livers (Figure 2, E and F). Therefore, using p-S6 and p-4EBP1 as mTORC1 activation markers, our results demonstrate that (a) loss of GS can further activate mTORC1; (b) GS plays an essential role in p-mTOR phosphorylation at S2448, whose biological relevance and underlying mechanisms remain to be determined; and (c) the use of p-mTOR S2448 as an mTORC1 activation marker needs to be further investigated, as previously cautioned (28), in mouse liver studies. These notions were further supported by our observation that GS^lo^p-4EBP1^hi^p-mTOR S2448^lo^ cells appeared to accumulate along the progression of WT tumors (Supplemental Figure 2C).

Our findings point to a role of Glu-derived NEAAs in activating mTORC1 and promoting HCC development upon β-catenin activation and loss of *GLUL*. β-Catenin activation led to an inhibition of the urea cycle and decreased urea production, leading to an accumulation of ammonia. GS ablation, as expected, blocked Gln synthesis and reciprocally increased the Glu pool. This is reminiscent of previous findings in breast cancer, where increased GDH activity was found to enhance the assimilation of intratumoral ammonia waste into Glu and the subsequent production of amino acids to enhance tumor growth (19). Our in vivo ^15^N-NH_4_Cl and ^13^C-U-glucose i.p. bolus tracing showed a rapid increase in ^15^N- and ^13^C-labeled Glu and Glu-derived Ala and Asp (Figure 6, H and J). While we also observed an increasing trend in the pool size of Gly, Pro, Asn, Met, Thr, His, and Lys (Figure 6, H and I) and cannot rule out the possibility that they may contribute to tumor growth, these amino acids were likely products of secondary reactions since there was no prominent incorporation of labeled ammonia. It is also possible that a backup accumulation of aKG contributes to mTORC1 activation or epigenetic alterations (57, 58). Nonetheless, our cell culture results strongly indicate that Glu-derived NEAAs have a major contribution to mTORC1 activation and cell growth (Figure 7 and Supplemental Figure 6). Indeed, while much attention has focused on mTORC1 activation by Leu, Arg, Met, and Gln (35–37), other amino acids, including Ala, have been reported to activate mTORC1 (39–41). It is particularly important to note that although Gln may be sensed by cells to directly activate mTORC1 (59), its mTORC1-stimulating function requires its conversion into NEAAs, including Ala (41). Therefore, it is not entirely surprising that NEAAs can activate mTORC1 in the context of reduced Gln production. It remains to be determined how NEAAs activate mTORC1. Ala has been shown to activate mTORC1 by facilitating the uptake of other amino acids or serving as a priming amino acid in an obligate 2-step mTORC1-activating mechanism (35, 39). Nonetheless, as GPT has been implicated in various cancers and therapeutics (60–64), our study shows it is an important mediator of HCC development and a therapeutic target (Figure 7, K–T). Our study also emphasizes the importance of maintaining nitrogen homeostasis, such as by modulating dietary nitrogen content and intestinal flora, as potential strategies for HCC prevention and treatment.

## Methods

### Chemicals.

The following were purchased from Sigma-Aldrich: L-Ala (A7469), L-Asn (A4159), L-Asp (A7219), L-Glu (G8415), L-Ser (S4311), L-Gln (G8540), L-Arg (A8094), L-Cys (C7352), L-Pro (P5607), NH_4_Cl (A9434), dmKG (349631), L-cycloserine (C1159) at 250 μM, DEN (N-0756), and GDH (G2626). Also used were rapamycin (LC Laboratories, R-5000; 2 mg/kg for in vivo, 10 μM for in vitro); β-CLA (Santa Cruz Biotechnology, sc-291972) at 250 μM for cell culture, and 20 mg/kg for in vivo experiments; and DL-Cyc (MCE, HY-W008440) at 250 μM. Direct Red 80 stable isotope tracers were purchased from Cambridge Isotope Laboratories: ^15^N-NH_4_Cl (NLM-467-PK), U-^13^C-D-glucose (CLM-1396-1), ^15^N-NH_4_OAc (NLM-177-PK), α-^15^N-L-Gln (NLM-1016-PK), amide-^15^N-L-Gln (NLM-557-PK), and U-^13^C-aKG (CLM-2411-PK).

### Constructs.

The following plasmids were used for mouse injection and cell culture experiments: pT3-EF1aH c-Met (Addgene, 86498, human c-Met or hMet), pT3-EF1aH YAP S127A (Addgene, 86497, with Flag-tag), pCMV/SB10 transposase (Addgene, 24551), pX330-U6-Chimeric_BB-CBh-hSpCas9 (pX330) (Addgene, 42230), and pLentiCRISPRv2 (Addgene, 52961). pT3-EF1α-ΔN90-β-catenin (with Myc-tag) and sgAXIN1.1 were previously described (24). sgRNA against mouse GPT1 (sg*Gpt1*: TCCAAGGCACGTTGCACGAT) was constructed in the pX330 plasmid. To delete *GLUL* in human liver cancer cells, we cloned an sgRNA against human *GLUL* (sg*GLUL*: GCGCTGCAAGACCCGGACCC) into the pLentiCRISPRv2 vector. Additional plasmids, including human si*GOT1*, si*GPT*, si*PSAT1*, and si*TAT*, were purchased from Horizon. All plasmids for in vivo studies were purified using the Plasmid Maxi Kit (Qiagen, 12163).

### Antibodies.

Antibodies against the following proteins were purchased from the indicated sources: GS (Sigma-Aldrich, G2781; 1:1,000 for IB, 1:500 for IHC; BD Biosciences, 610517, 1:800 for IHC and IF), p-S6K1 T389 (Cell Signaling Technology [CST], 9234s; 1:500 for IB), S6K1 (CST, 9202; 1:1,000 for IB), p-S6 S235/236 (CST, 4858; 1:2,000 for IB, 1:200 for IHC), p-S6 S240/244 (CST, 2215; 1:1,000 for IB), S6 (CST, 2217; 1:1,000 for IB), p-4EBP1 T37/46 (CST, 2855; 1:1,000 for IB; 1:3,200 for IHC, 1:200 for IF), p-4EBP1 S65 (CST, 9451; 1:1,000 for IB), 4EBP1 (CST, 9644; 1:1,000 for IB), p-mTOR S2448 (CST, 2976; 1:100 for IHC and IF), p-CAD S1859 (CST, 12662; 1:500 for IB), p-Akt S473 (CST, 4060; 1:1,000 for IB), Akt (CST, 9272; 1:1,000 for IB), p-AMPKa T172 (CST, 2535; 1:1,000 for IB), p62 (CST, 8025; 1:1,000 for IB), LC3B (CST, 2775; 1:1,000 for IB), β-catenin (BD Biosciences, 610153; 1:2,000 for IB; Thermo Fisher Scientific, 13-8400; 1:400 for IF; CST, 8480 1:100 for IF), β-actin (CST, 4970; 1:1,000 for IB), p-histone H2A.X (S139) (CST, 9718; 1:1,000 for IB), arginase I (CST, 93668; 1:1,000 for IB, 1:100 for IHC and IF), p-eIF2α (S51) (CST, 3398; 1:1,000 for IB), p-Erk1/2 (T202/Y204) (CST, 4370; 1:1,000 for IB), p-p38 MAPK (T180/Y182) (CST, 4511; 1:1,000 for IB), CPS1 (Santa Cruz Biotechnology, sc-376190; 1:1,000 for IB, 1:200 for IHC, 1:100 for IF), β-tubulin (Proteintech, 66240-1-Ig; 1:2,000 for IB), GAPDH (CST, 97166; 1:1,000 for IB), PCNA (CST, 13110; 1:8,000 for IHC), GOT1 (Proteintech, 14886-1-AP; 1:1,000 for IB), GPT1 (Proteintech, 16897-1-AP; 1:1,000 for IB), OTC (Proteintech, 26470-I-AP; 1:1,000 for IB, 1:100 for IF), ASS1 (CST, 70720; 1:1,000 for IB, 1:1,600 for IF), ASL (Novus, NBP1-87462; 1:1,000 for IB), GPT1 (Proteintech, 16897-1-AP; 1:1,000 for IB), TAT (Santa Cruz Biotechnology, sc-376292; 1:1,000 for IB), PSAT1 (Proteintech, 10501-1-AP; 1:1,000 for IB), GLUD1 (Proteintech, 14299-1-AP; 1:5,000 for IB), Hsp70 (Abcam, ab2787; 1:100 for IHC), α-SMA (Abcam, ab124964; 1:1,000 for IHC), CYP2E1 (Thermo Fisher Scientific, PA5-52652; 1:300 for IHC), F4/80 (CST, 70076; 1:200 for IHC), goat anti-mouse–Alexa Fluor 488 (Life Technologies, 11001), and goat anti-rabbit–Alexa Fluor 594 (Life Technologies, 11012).

### Mouse experiments.

*Glul^fl/fl^* (mixed 129/Ola and C57BL/6) (22) and Alb-Cre (The Jackson Laboratory, strain 003574, C57BL/6) were used. Given that HCC/liver cancer is a sexually dimorphic disease with preponderance in males, male mice were used for in vivo tumor-related studies.

### HTVI and related experiments.

HTVI was performed as previously described (27, 65). Briefly, 20 μg of pT3-EF1aH c-Met and pT3-EF1α-ΔN90-β-catenin or pT3-EF1aH YAP S127A and pT3-EF1α-ΔN90-β-catenin along with 1.6 μg pCMV-SB10 transposase at a ratio of 25:1 was diluted in 2 mL of Ringer’s solution, filtered through a 0.22 μm filter (Millipore, GSWP04700), and injected into the lateral tail vein in 5–7 seconds. For c-Met/sgAxin1–induced tumorigenesis models, mice received 40 μg pX330 sgAxin1.1 mixed with 20 μg pT3-EF1α-c-Met (human) along with 0.8 μg pCMV-SB10 in 2 mL of Ringer’s solution. For rapamycin treatment experiments, 7-week-old male mice were first injected with c-Met/Δ90-β-catenin/pCMV-SB10 plasmids, and then half of the mice were i.p. injected with rapamycin (2 mg/kg, 3 times per week), and the other half were i.p. injected with vehicle (5% Tween 80, 5% PEG 400 in 1× PBS; 3 times per week). For in vivo GPT1 inhibition (sg*GPT1*) experiments, 7-week-old *Glul*-KO male mice were injected with c-Met/ΔN90-β-catenin/pCMV-SB10 plasmids, together with pX330 sgCtrl or pX330 sgGPT1 plasmids, via SB-HTVI. For β-CLA treatment, 7-week-old *Glul*-KO male mice were first injected with c-Met/Δ90-β-catenin/pCMV-SB10 plasmids, and then half of the mice were i.p. injected with either PBS or β-CLA (20 mg/kg, 3 times per week). For the tail vein injection of adenoviral-Cre to knock out GS in adult mice, adenoviral CMV-Cre (2 × 10^–9^ PFU or 9 × 10^–12^ particles/mL; University of Iowa Vector Core) and adenoviral CMV-GFP (2 × 10^–9^ PFU) were injected into male mice 1 week after the c-Met/Δ90-β-catenin/pCMV-SB10 plasmid injection.

### Carcinogen-induced mouse tumor models.

For the DEN/PB-induced HCC model, 14-day-old male mice were injected with DEN (5 mg/kg; Sigma-Aldrich, N-0756) via i.p. injection and then fed 0.05% PB in drinking water 7 days later (66, 67). Livers from DEN/PB-treated mice were harvested after 8 months. For the DEN/HFD-induced HCC model, 14-day-old male mice were i.p. injected with DEN (15 mg/kg) and then fed an HFD (42% kcal from fat; Envigo, TD.88137) 7 days later. Livers from DEN/HFD-treated mice were collected after 6 months. For acute DEN genotoxicity, 6- to 8-week-old male mice were i.p. injected with vehicle or DEN (200 mg/kg) and sacrificed after 24 hours. Unless specified otherwise, all mouse experiments, including the sampling, were performed by removing chow at 8 to 9 am and performing the experiments at approximately 2 pm to avoid the potential influence of circadian rhythm and feeding status.

### TMAs.

Deidentified clinical TMAs were purchased from Shanghai WellBio technology (ZL-LVC1607, ZL-LVC1608, and ZL-LVC1801), Bioaitech (D2000601), and US Biomax, Inc (BC03116a and BC03117).

### Statistics.

IB images were quantified by Odyssey Infrared imaging system software (version 3.0, LI-COR Biosciences) or ImageJ software (NIH). All statistical analyses were performed with more than 3 independent biological replicates for cell culture studies or with the indicated numbers of animals for mouse studies. Data were analyzed and graphed using GraphPad Prism 9.3.0, and all summary data are presented as mean ± SD. For analysis between 2 groups, a 2-tailed *t* test was applied. One-way ANOVA with Tukey’s multiple comparisons test was used for comparisons between more than 2 groups. For the Kaplan-Meier survival plots, statistical significance was measured using the log-rank (Mantel-Cox) test. Correlations between GS and urea cycle–related enzymes in Figure 9B was analyzed using Pearson’s correlation. The results were considered significant when *P* was less than 0.05: *P < 0.05, **P < 0.01, ***P < 0.001, ****P < 0.0001. NS, not significant.

### Study approval.

All mouse experiments were performed in compliance with the Institutional Animal Care and Use Committee guidelines at Rutgers University.

### Data availability.

The data sets generated in this study are available in TCGA (https://portal.gdc.cancer.gov/) and the NCBI Gene Expression Omnibus GEO (https://www.ncbi.nlm.nih.gov/gds; GEO GSE201560). Please see Supplemental Methods for other details.

## Author contributions

WD and WXZ conceived the study. WD and JS performed most of the experiments. JY, AJB, SM, HQD, Yujue W, and KK performed experiments. JYG, WXD, AV, and XC contributed methodology and experimental resources. JY, LZ, and Yongbo W performed data analysis. XC and XS helped design experiments and provided technical assistance. SG performed data analysis and wrote the manuscript. WXZ supervised the study and wrote the manuscript. WD and JS are listed as co–first authors; WD is listed first because of his contributions to conceiving the study. All authors have read and approved the final version of the manuscript.

## Supplementary Material

Supplemental data

## Version 1 10/18/2022

In-Press Preview

## Version 2 12/15/2022

Electronic publication
