# Supplementary material for: Glutamine synthetase limits β-catenin–mutated liver cancer growth by maintaining nitrogen homeostasis and suppressing mTORC1
Source: J Clin Invest. 2022 Dec 15;132(24):e161408. doi: 10.1172/JCI161408 (PMC9754002; doi:10.1172/JCI161408)
Supplement: Supplemental data [file jci-132-161408-s207.pdf]

Supplementary Figures

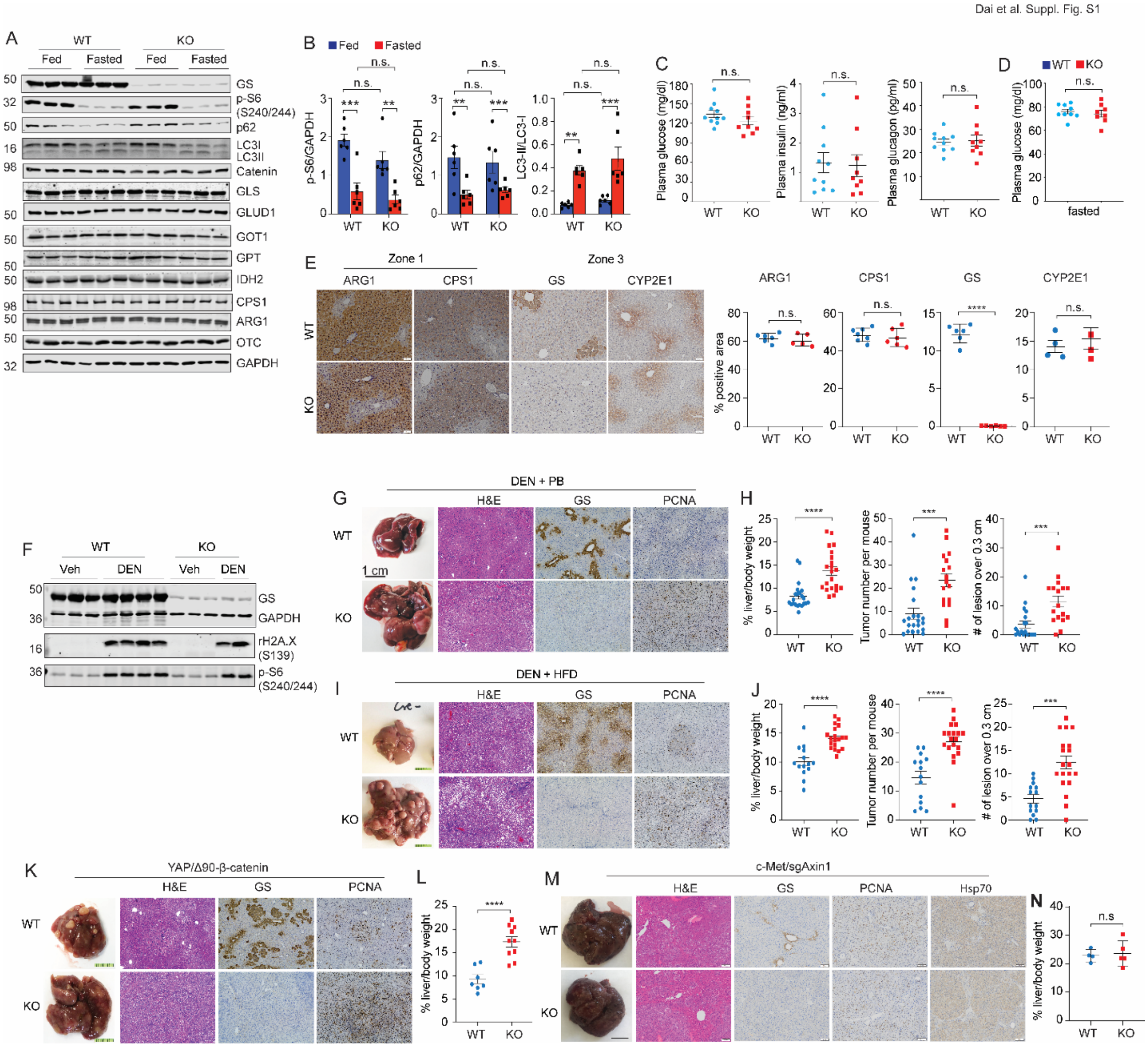

Suppl. Fig. S1. Hepatic GS ablation promotes liver cancers induced by DEN and YAP/Δ90-*β*-catenin but not c-Met/sgAxin1. (A and B) Seven-week-old male and female WT and KO mice were fasted for 16 h, and then half of the mice were refed for 2 h (Fed), while the other half remained fasted (Fasted). The indicated proteins were probed by immunoblotting **(A)**. Quantifications of the ratios of p-S6 S240/244/GAPDH, p62/GAPDH, and LC3II/LC3I are shown **(B)**. ****p<0.0001 (One-way ANOVA). **(C)** Plasma glucose, insulin, and glucagon levels were measured in 6 h-fasted mice (n=9-10). **(D)** Plasma glucose levels were measured in 16 h-fasted mice (n=8-9). (E) Seven-week-old WT and KO male mice livers were stained for zonation markers ARG1 and CPS1 for zone 1, and GS and CYP2E1 for zone 3, by IHC. Positive stained areas were quantified by Image J. 4-6 randomly selected areas in each group were chosen.

Note that GS ablation does not affect the liver zonation. **(F)** Mice were IP-injected with vehicle or DEN (200 mg/kg), and livers were harvested after 24 h. Immunoblotting shows similar DNA damage (rH2A. X) and p-S6 (S240/244) levels in WT and KO livers. (**G and H)** Fourteen-day-old WT and KO male mice (n=21) were IP injected with DEN (5 mg/kg) and then fed 0.05% phenobarbital (PB) in drinking water 7 days later. Livers from DEN/PB-treated mice were harvested after 8 months. Representative gross, H&E, and IHC staining images are shown **(G)**. The liver/body weight ratio, numbers of total visible tumors, and numbers of tumors >0.3 cm in diameter were plotted (n=17-22) **(H)**. (**I and J)** Fourteen-day-old WT and KO male mice (n=17) were IP injected with DEN (15 mg/kg) and then fed a high-fat diet (HFD, 42% kcal from fat) 7 days later. Livers were collected after 6 months. Representative gross, H&E, and IHC staining images are shown **(I)**. The liver/body weight ratio, numbers of visible tumors, and numbers of tumors >0.3 cm in diameter were plotted (n=14-17) **(J)**. **(K and L)** Seven-week-old WT and KO male mice (n=10) were coinjected with YAP/ΔN90-β-catenin/pCMV-SB10 plasmids via SB-HTVI. Livers were harvested 9.6 weeks later. Representative gross, H&E, and IHC images are shown **(K)**. The liver/body weight ratio was plotted (n=7-10) **(L)**. **(M and N)** Seven-week-old WT and KO male mice (n=5) were coinjected with c-Met/sgAxin1/pCMV-SB10 plasmids via SB-HTVI, and livers were harvested 8 weeks later. Representative gross, H&E, and IHC images are shown **(M)**. Liver/body weight ratios are shown **(N)**. Paired t-test was used to compare two groups. n.s: nonsignificant. **p<0.01, ***p<0.001, ****p<0.0001.

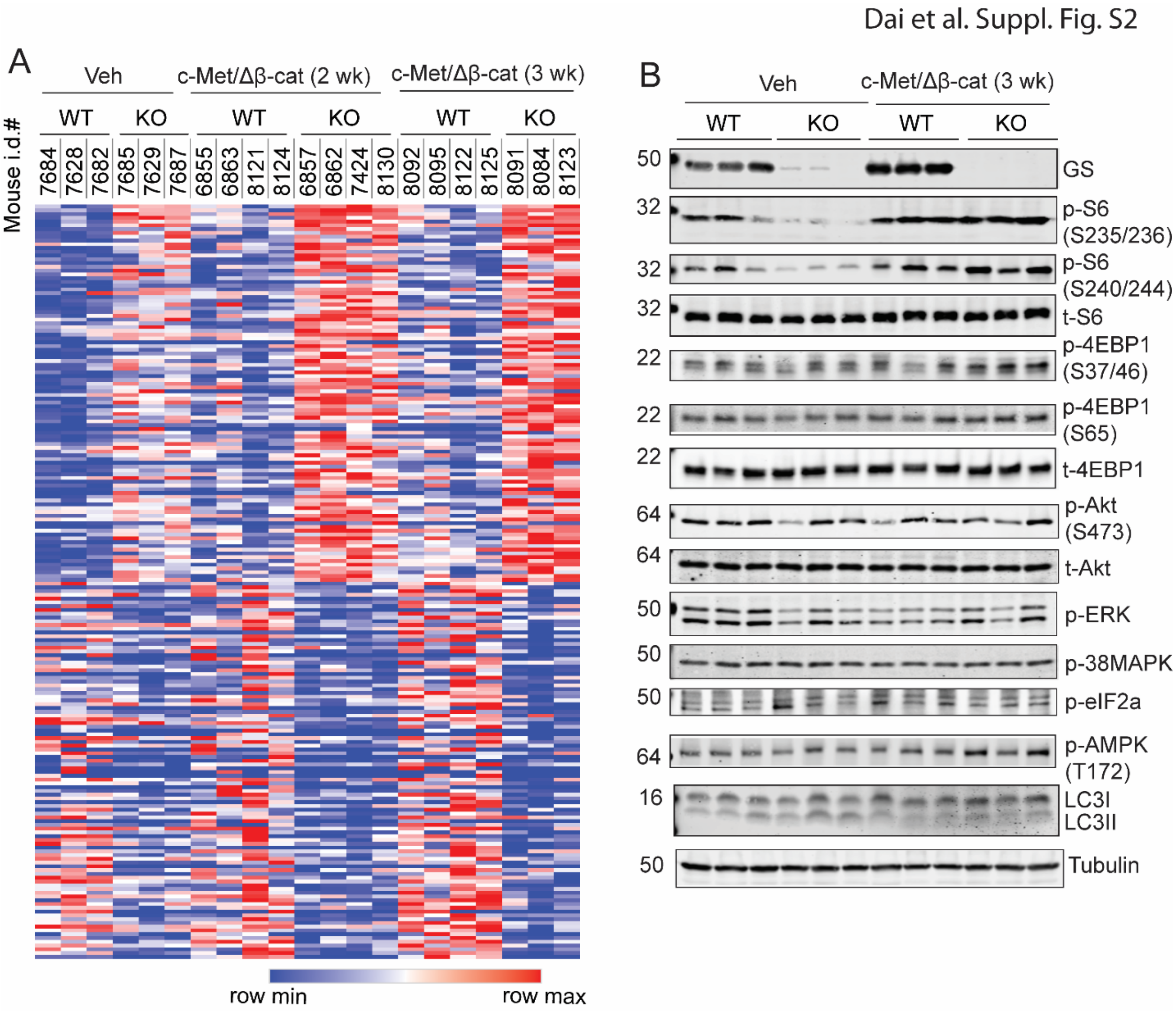

Suppl. Fig. S2. Hepatic GS ablation elevates mTORC1 activation in mouse livers harboring c-Met/Δ90-*β*-catenin. Seven-week-old WT and KO male mice were injected with vehicle (Veh) or c-Met/ΔN90-*β*-catenin/SB10 plasmids via SB-HTVI. **(A)** Livers were harvested at 0, 2, or 3 weeks after oncogene injection and were subjected to RNA-seq. Heatmap of relative expression indicates significantly altered gene expression patterns genome wide. **(B)** Immunoblotting of liver lysates that were harvested 3 weeks after oncogene injection.

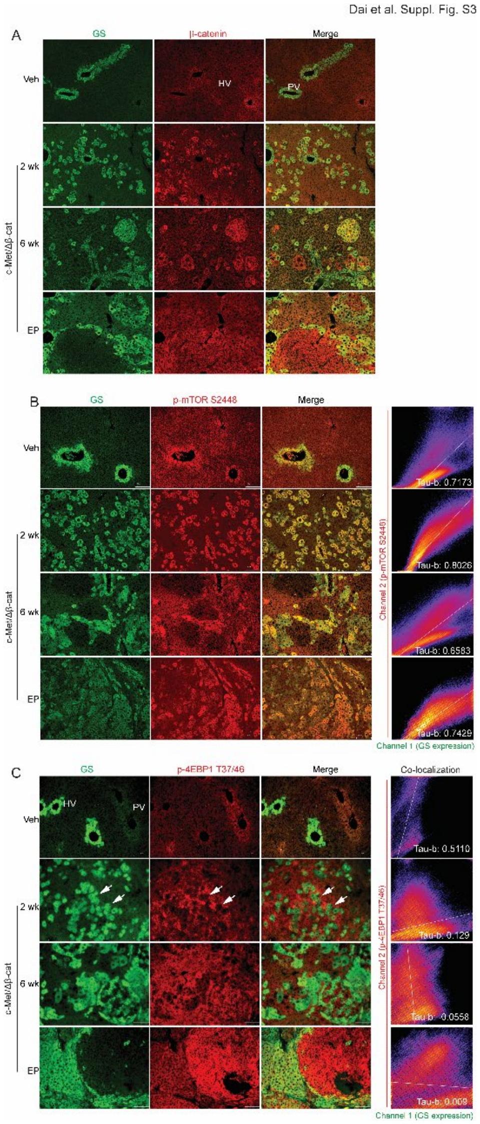

Suppl. Fig. S3. Characterization of mTORC1 signaling during HCC progression in WT livers. (A) Co-IF for GS (green) and β-catenin (red) during tumor progression in livers expressing c-Met/ΔN90-*β*-catenin. Note that some b-catenin-positive cells were low for GS, whose population progressively expanded during tumor progression. **(B and C)** Co-IF for GS (green)/p-mTOR S2448 (red) **(B)** or GS (green)/p-4EBP1 T37/46 (red) **(C)** in WT mouse livers harvested at 0, 2, 6, and 9 (endpoint, EP) weeks after oncogene injection (n=3). Representative images are shown. Fluorescence images were subjected to colocalization analyses and were judged positively correlated when Kendall's tau-b>0.5. Note that GS inversely correlates with p-4EBP1 T37/46 and positively correlates with p-mTOR S2448. Additionally, note that some tissue areas/cells become GS^{low}, p-mTOR $S2448^{low}$, and p-4EBP1 $T37/46^{high}$ during tumor progression. PV: portal vein; HV: hepatic vein. White arrowheads highlight cells with induced GS expression yet decreased p-4EBP1 T37/46.

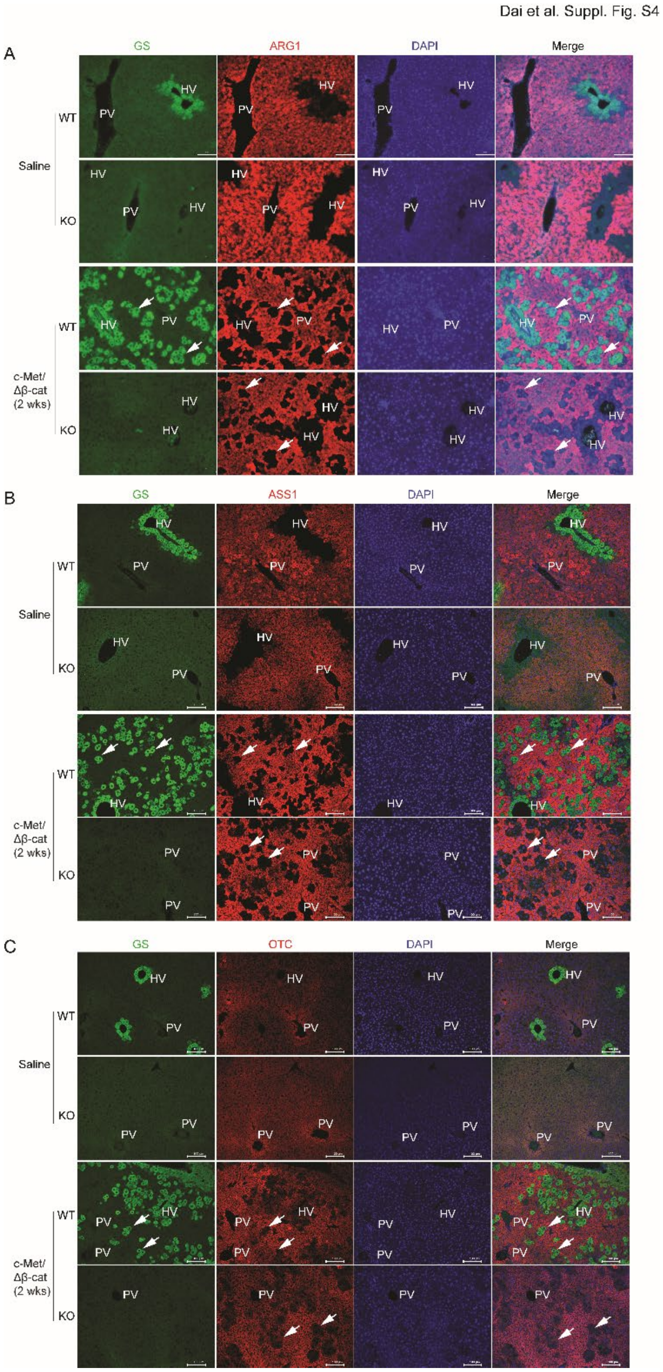

Suppl. Fig. S4. c-Met/Δ90-*β*-catenin suppresses UCEs. Seven-week-old WT and KO male mice were injected with saline (vehicle) or c-Met/Δ90-*β*-catenin/SB10 plasmids via SB-HTVI, and livers were harvested 2 weeks after the injection. Liver sections were costained for GS (green)/ARG1 (red)/DAPI (blue) **(A)**, GS (green)/ASS1 (red)/DAPI (blue) **(B)**, and GS (green)/OTC (red)/DAPI (blue) **(C)**. Representative images are shown. The portal vein (PV, GS-low and UCE-high) and hepatic vein (HV, GS-high and UCE-low) are shown. Arrows point to exemplified cells showing an inverse correlation between GS and UCEs in the WT livers.

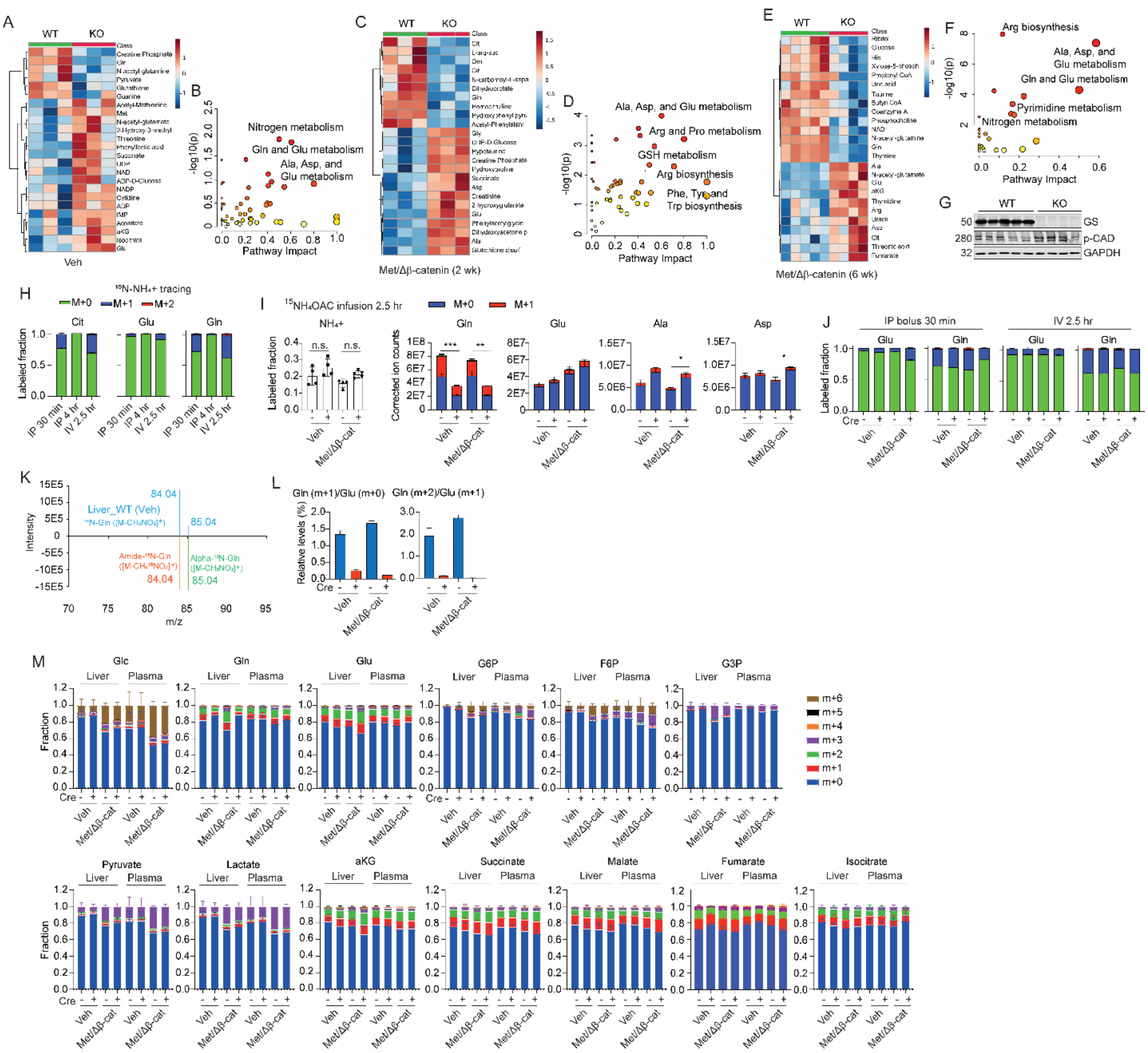

Suppl. Fig. S5. Metabolomics characterization of WT and KO livers upon oncogene activation. (A-G) Seven-week-old WT and KO male mice were injected with vehicle (Veh) or c-Met/ΔN90-*β*-catenin/SB10 plasmids via SB-HTVI. Liver tissues were collected at 0, 2, and 6 (endpoint of oncogene-injected KO mice) weeks. Polar metabolites were extracted and subjected to LC-MS. A summary of the top 25 altered metabolites (t-test/ANOVA) and metabolic pathways (MetaboAnalyst) is shown **(A-F)**. Phospho-CAD was probed by WB in liver tissues harvested 6 weeks post oncogene injection **(G)**. **(H and I)** WT and KO mice injected with oncogenes for 2 weeks were subjected to IP injection of 5 mmol/kg $^{15}N\text{-}NH_4Cl$ for 30 min, 4 h, or IV infusion with $^{15}N\text{-}NH_4OAc$ (0.3 M in saline at 0.1 μl/g/min) for 2.5 h. Labeled fractions of Cit, Glu, and Gln in healthy WT mice are shown. Note that IP 30 min and IV 2.5 h gave similar

labeling efficiency (**H**). Circulating ammonium was measured by LC/MS after a GDH-mediated conversion of ammonium and 13C-aKG into Glu. Fraction of enriched ^{15}N-ammonium was compared among the 4 groups of mice. Labeled efficiency for the NEAAs in the liver is also shown (**I**). **(J)** Glu and Gln labeling efficiency in IP 30 min and IV 2.5 h mice are shown for all 4 groups of mice. Note that while IP 30 min shows increased Glu and decreased Gln labeling in oncogene-expressing KO livers, the differences are diminished in the 2.5 h infusion mice, suggesting a mask of metabolism in other tissues. **(K)** The MS/MS spectra of ^{15}N-Gln. Fragment ions 84.04 and 85.04 were generated from $^{15}N_1$-Gln. The top spectrum (blue) is from the liver sample. The bottom panel shows the combined spectra of amide-$^{15}N_1$-Gln (84.04, red) and alpha-$^{15}N_1$-Gln (85.04, green). **(L)** The ratios of Gln (m+1)/Glu (m+0) and Gln (m+2)/Glu (m=1) are similar in all WT livers and much decreased in all KO livers, indicating that the enrichment of labeled Gln in WT livers is from GS activity, which is lost in the KO livers. **(M)** Mice injected with oncogenes for 2 weeks were IP injected with 5 mmol/kg ^{13}C-U-glucose. Liver tissues and plasma were collected and snap-frozen 30 min after IP injection. Polar metabolites were extracted and subjected to LC-MS. Labeled fractions of key metabolites related to glucose metabolism, the TCA cycle, and Gln metabolism in both liver and plasma were analyzed and are shown. The results are expressed as the means ± SEM (n=3-5 in each group).

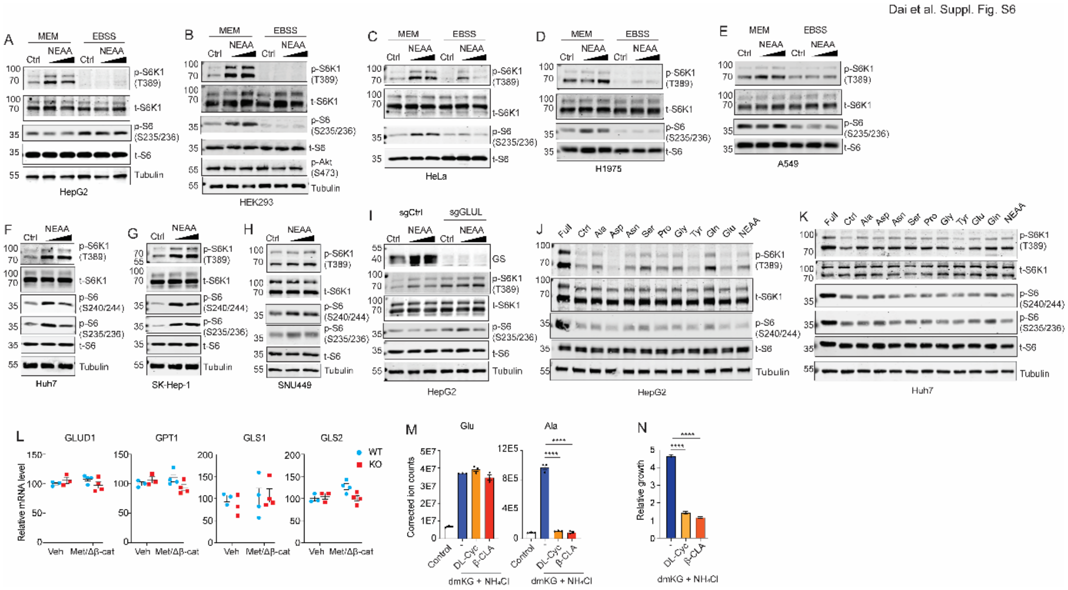

Suppl. Fig. S6. mTORC1 activation induced by NEAAs in other liver cancer cells. (A-E) Indicated cell lines were prestarved in MEM or EBSS for 8 h and then stimulated for 30 min with 1× and 2×NEAA mix. mTORC1 activity was analyzed by immunoblotting. **(F-H)** mTORC1 activity was determined in Huh7 cells prestarved in EBSS for 24 h **(F)**, in SK-Hep-1 cells prestarved in MEM for 4 h **(G)**, and in SNU449 cells prestarved in MEM for 8 h **(H),** then stimulated in 1× and 2×NEAA mix for 30 min. **(I)** HepG2 cells stably expressing CRISPR v2 (sgCtrl) and sg*GLUL* were prestarved in MEM for 8 h and then stimulated for 30 min with 1× and 2×NEAA mix. **(J)** HepG2 cells were prestarved in MEM for 8 h and left unstimulated (Ctrl) or stimulated for 30 min with 0.3 mM of indicated individual amino acid or 1×NEAA mix. Cells cultured in full medium (EMEM with 10% FBS) were used as a positive control (Full) for mTORC1 signals. **(K)** Huh7 cells were prestarved in EBSS for 8 h and then stimulated for 30 min with 1 mM of indicated individual amino acid or 1×NEAA mix. (**L**) WT (Cre^-) and KO (Cre^+) mice with vehicle or c-Met/ΔN90-*β*-catenin expression for 2 weeks were harvested. Expression levels of indicated proteins were determined by RNA-seq. **(M)** Hep3B cells pre-starved in MEM medium for 8 h and pre-treated with vehicle, DL-Cyc, or *β*-CLA for 1 h, then left untreated (control) or stimulated with 1.5 mM dmKG + 4 mM NH_4Cl for 2 h. Glu and Ala level was determined by LC-MS. The mean ± SEM of experiments (three repeats) is shown. **(N)** Hep3B cells were cultured in MEM medium supplemented with 0.2 mM Gln, 1.5 mM dmKG, and 4 mM NH_4Cl, together with vehicle (control), DL-Cyc, or *β*-CLA. Relative cell growth was determined 72 h later. The mean ± SEM of experiments (three repeats) is shown. ****p<0.0001 (One-way ANOVA).

Supplementary Methods

Plasma and liver sample collection: At the end of the *in vivo* experiments, mice were weighed, and plasma and liver tissues were harvested. Unless described otherwise, all mouse experiments, including sampling, were performed by removing chow at 8-9 am and performing the experiments at approximately 2 pm to avoid the potential influence of circadian rhythm and feeding status. Whole blood from the heart was collected into heparinized tubes, gently mixed, and stored on ice. Cells were removed from plasma by centrifugation for 15 min at 2,000 × *g* at 4°C. The resulting supernatant was immediately transferred into a clean tube, aliquoted, snap-frozen in liquid nitrogen, and stored at -80°C until further analysis. Liver tissue samples were snap-frozen in liquid nitrogen with a precooled Wollenberger clamp. The frozen sample was broken into smaller pieces, stored in foil and kept at -80°C for LC-MS, RNA-seq/RT-PCR, and immunoblotting analysis. Liver tissues were also fixed in 10% formalin in PBS overnight at 4°C and then processed and embedded in paraffin for histology, IHC, and IF analyses. Plasma glucose, glucagon, and insulin were analyzed at Vanderbilt Hormone Assay & Analytical Services Core (Vanderbilt University Medical Center, Tennessee).

Isolation of tumor interstitial fluids (TIF): Interstitial fluid was isolated as described (6). Briefly, tumors >100mm^3 were excised, briefly rinsed at room temperature with 1 × PBS, and blotted dry on filter paper. Tumors were placed onto 20 µm Spectra/Mesh Woven Nylon filters (Spectrum Labs, Waltham, MA, #148134) affixed to 50 mL conical tubes and centrifuged at 106 x *g* at 4°C for 10 mins. TIF was then collected from the conical tube, snap-frozen in liquid nitrogen, and stored at -80°C until further analysis.

Ammonia measurement: Ammonia levels in plasma and TIF were measured using a colorimetric assay (Abcam, #ab83360) following the manufacturer's instructions. Briefly, TIF samples were diluted 10 times using ammonia assay buffer. Five µl of plasma and prediluted TIF samples (adjusted volume to 50 µl with ammonia assay buffer), standard dilutions (50 µl), and background control samples were loaded into a 96-well plate in duplicate. Then, 50 µl of ammonia reaction mix or background reaction mix was added. The plate was incubated at 37 °C for 60 min protected from light and measured at OD 570 nm in a microplate reader.

***In vivo* stable isotope labeling:** For ^{15}N-NH_4Cl *in vivo* tracing experiments, mice were IP-injected with 5 mmol/kg ^{15}N-NH_4Cl (1.25 mol/L, 100 µl/25 g body weight) in Hanks' Balanced Salt solution (HBSS, Sigma H6648). Liver tissues were collected and snap-frozen 30 min and 4 h, respectively, after IP injection. Polar metabolites were extracted and subjected to LC-MS. For jugular vein catheterization, the procedure was modified from work previously described (7). In brief, mice were anaesthetized using isoflurane carried by 2% oxygen, followed by the placement of a central venous catheter (SAI Infusion Technologies) into the right jugular vein. A minimal amount of blood was carefully withdrawn to verify catheter patency. The proximal end of the catheter was then tunneled subcutaneously, exited between the shoulder blades and properly secured with a catheter button. Catheterized mice were connected to an infusion pump. ^{15}N-NH_4OAc (300 mM) was dissolved in sterile saline and infused at a rate of 0.1 µl/g/h for 2.5 h. Mice were sacrificed, and liver tissues were collected and analyzed by LC-MS. For *in vivo* U-$^{13}C_6$-D-glucose tracing experiments, mice were IP injected with 5 mmol/kg ^{13}C-glucose. Liver tissues and plasma were collected and snap-frozen 30 min later. Polar metabolites were extracted and measured by LC-MS.

Measurement of enriched ^{15}N-ammonium: The method for measurement of enriched ammonium was adjusted from previously described (8). Briefly, 2 µl of GDH (Sigma G2626) enzyme solution (500 units/ml), 5 µl of NADH solution (25 mM), 5 µl of ^{13}C-U-aKG solution (10 mM) and 5 µl of Tris-HCl (1 mM, pH 8.8) were mixed. 25 µl of plasma sample and 25 µl of pure water were added and incubated at 35°C for 30 min. After 30 min, 950 µl 40/40/20 MeOH/CAN/H2O with 0.5% formic acid was added to quench the reaction for later LC/MS analysis. $^{13}C_5$;^{15}N-labeled glutamate was detected as labeled ammonium, while $^{13}C_5$-labeled glutamate was detected as unlabeled ammonium.

Cell culture: Hep3B (ATCC, HB-8064), Hep G2 (ATCC, HB-8065), SK-Hep-1 (ATCC, HTB-52), and SNU-449 (ATCC, CRL-2234) cells were obtained from ATCC. Huh7 cells were obtained from Creative Bioarray (CSC-C9441L). Hep3B, HepG2, and SK-Hep-1 cells were cultured in Eagle's minimum essential medium (EMEM) (ATCC, ATCC® 30-2003™) supplemented with 10% bovine serum (Fetal Clone III, HyClone), 100 units/ml penicillin and 100 µg/ml streptomycin following ATCC's instructions. SNU449, H1975, and A549 cells were cultured in RPMI-1640 medium (Sigma, R8758-500 mL) supplemented with 10% FBS and 1% P/S. HEK293, HeLa, and Huh7 cells were cultured in Dulbecco's modified Eagle's medium (DMEM, Thermo Fisher, #11965-118) supplemented with 10% FBS and 1% P/S. Lipofectamine 2000 (Invitrogen,

#11668019) and Opti-MEM Reduced Serum Medium (Gibco™, #31985070) were used for transfection. The pLentiCRISPRv2 and pLenti-sg*GLUL* plasmids were sequentially introduced into Hep3B and HepG2 cells. Briefly, lentiviruses were produced in HEK293T cells by transfecting plasmids and packaging plasmids (psPAX2 and VSV-G) with Lipofectamine 2000. Lentivirus-containing medium was harvested at 24, 48, and 72 h and filtered through a 0.22 μm filter (Millipore, GSWP04700). Hep3B and HepG2 cells were infected with virus-containing medium 3 times prior to puromycin selection. Lipofectamine™ RNAi MAX (ThermoFisher, #13778-100) was used for siRNA-related experiments.

To check non-essential amino acid (NEAA)-induced effects on mTORC1 activation, cells were pre-starved in Minimum Essential Medium (MEM, ThemoFisher, #11090081) supplemented with 5% dialyzed FBS (GEMINI, #100-108), 100 units/ml penicillin and 100 μg/ml streptomycin or Earle's Balanced Salt Solution (EBSS, ThemoFisher, # 24010043) supplemented with 5% dialyzed FBS, 1% P/S for 4 or 8 h and then stimulated for 30 mins with 1× or 2×NEAA mix (Gibco™ MEM Non-Essential Amino Acids Solution, ThermoFisher, #12084947). For ammonia-induced effects on mTORC1 activation, Hep3B cells were pre-starved in MEM medium for 8 h and stimulated for 24 h by adding various concentrations of NH_4Cl. At the end of the stimulations, the cells were washed twice with cold PBS, and an appropriate volume of lysis buffer (with protease inhibitor) was added.

To investigate the effect of NEAAs on cell proliferation, cells were cultured in MEM supplemented with 0, 0.2, or 0.5 mM Gln and supplied with no (control) or 1×NEAA mix or 1 mM individual amino acids for 0-3 days. Cells cultured in MEM medium without supplement were used as a negative control; cells cultured in full medium were used as a positive control. To compare ammonia-induced cell proliferation in Hep3B sgCtrl and sg*GLUL* cells, Hep3B sgCtrl and sg*GLUL* cells were cultured in MEM medium containing 0 or 0.2 mM Gln and supplied with no (control) or 1.5 mM dimethyl 2-oxoglutarate + 4 mM NH_4Cl for 0-3 days.

To investigate GPT inhibition-induced effects on mTORC1 activation, Hep3B sgCtrl and sg*GLUL* cells were transfected with GOT1, GPT1, TAT, PSAT1, and MIX4 siRNAs for 48 hours using Lipofectamine™ RNAiMAX (ThermoFisher, #13778-100) and then cultured in MEM for acute stimulations or cell proliferation experiments. siMIX4 contains GOT1, GPT1, TAT, and PSAT1 siRNAs at a 1:1:1:1 ratio. For GPT inhibitor-related *in vitro* experiments, Hep3B sgCtrl and sg*GLUL* cells were pre-starved in MEM medium for 7 h, then pre-treated with L-cycloserine

(L-Cyc, Sigma C1159) at 250 μM; DL-cycloserine (DL-Cyc, MCE HY-W008440) at 250 μM, or *β*-chloro-L-alanine (*β*-CLA, Santa Cruz Biotechnology sc-291972) at 250 μM for 1 h.

Immunoblotting: Frozen livers (~100 mg) were homogenized on ice with a hand homogenizer in 10 times lysis buffer containing 150 mmol/L NaCl, 10 mmol/L Tris, 1 mmol/L EGTA, 1 mmol/L EDTA (pH 7.4), 100 mmol/L NaF, 4 mmol/L sodium pyrophosphate, 2 mmol/L sodium orthovanadate, 1% Triton X-100, 0·5% NP-40-Igepal and protease inhibitor (*Prote*CEASE™-50, *Plus* EDTA, G-Biosciences #786-334). Homogenates were centrifuged at 12,000 × *g* for 15 min at 4℃. The supernatant fractions were recovered and stored at -80℃. Lysates (15-25 μg of total protein) were subjected to SDS-PAGE and immunoblotting. All primary antibodies were incubated overnight at 4°C. Alexa Fluor-conjugated goat anti-rabbit (IRDye 800, Rockland #926-32211) or goat anti-mouse (Alexafluor-680, Life Technologies A21057) antibodies were used as secondary antibodies (1:20,000). Immunoblots were visualized by Infrared Fluorescence using the Odyssey Imaging System (LI-COR Biosciences) and quantified by Odyssey Infrared imaging system software (Version 3.0, LI-COR Biosciences) or ImageJ Software (NIH, Bethesda, MD).

Cells were carefully washed with two times cold PBS at the end of the stimulation period, and an appropriate volume of lysis buffer (with protease inhibitor) was added before the culture dishes were snap-frozen and stored at -80°C. Cells were scraped, collected, and lysed on ice for 30 min. Lysates were centrifuged at 12,000 × *g* for 15 min at 4°C. The supernatant fractions were recovered and stored at -80℃. Lysates (10-20 μg of total protein) were subjected to SDS-PAGE.

Histology and immunohistochemistry (IHC): Mouse liver tissues were fixed in 10% formalin in PBS overnight at 4°C and then processed and embedded in paraffin. Tissue sections of 4 μm thickness were cut from paraffin blocks and used for H&E, IHC, or Sirius Red staining. For IHC, paraffin-embedded liver sections were deparaffinized, rehydrated, and microwave heated for 15 min in 10 mM citrate buffer (pH 6.0) for antigen retrieval. Hydrogen peroxide (3%) was applied to block endogenous peroxidase activity. After 2.5 h of blocking with 10% goat serum, primary antibodies with the indicated dilutions or control IgG were applied and incubated overnight at 4°C. After washing, slides were incubated with biotinylated secondary antibody, and the streptavidin-biotin complex (Vectastain Elite ABC kit, Vector Laboratories) was applied, each for 30 min at room temperature with an interval washing. After being rinsed with PBS, the slides were immersed for 0.5-5 min (determined by observation under a microscope) in the coloring

substrate 3,3′-diaminobenzidine (DAB, Cell Signaling # 8059S) 0.4 mg/mL with 0.003% hydrogen peroxide, rinsed with distilled water, counterstained with hematoxylin, dehydrated, and coverslipped. H&E and IHC slides were examined using light microscopy (Eclipse Ci, Nikon Instruments) and photographed using a Keyence BX-Z microscope. For Sirius Red staining, paraffin-embedded liver sections were deparaffinized, rehydrated and stained with hematoxylin for 8 min, followed by staining in Picro Sirius Red (Direct Red 80, Sigma 365548; Picric acid, Sigma 197378) for 1 h. After washing in two changes of 0.5% acidified water, slides were dehydrated and coverslipped.

For Oil Red O staining, frozen liver sections were fixed in cold propylene glycol for 2 min and then incubated on slides in Oil Red O solution (Oil Red O stain kit, ab150678) for 8 min. Sections were differentiated in 85% propylene glycol for 1 min, rinsed on slides with 2 changes of distilled water, incubated in hematoxylin for 2 min and softly rinsed on slides in tap water and 2 changes of distilled water. Coverslip using an aqueous mounting medium. Images were visualized and scanned using a virtual/digital slice microscope (VS120, Olympus).

Immunofluorescence: Paraffin-embedded liver sections were deparaffinized, rehydrated, and microwave heated for 15 min in 10 mM citrate buffer (pH 6.0) for antigen retrieval and then permeabilized in 0.4% Triton X-100 for 10 min at RT. After washing 3 times with PBS, tissues were blocked in 10% animal serum and probed with primary antibodies at 4°C overnight. Sections were washed and labeled with the appropriate fluorescently conjugated secondary antibodies (goat anti-rabbit Alexa Fluor 594 #11012; goat anti-mouse Alexa 488 #11001, 1:500 dilution, Life Technologies) for 1 h at room temperature and then mounted with Pronlong Gold antifade reagent with DAPI (Invitrogen P36935). Images were captured using a Keyence BX-Z microscope. For TMA IF staining, slides were scanned using a Celigo 5C Imaging Cytometer.

Colocalization analysis: Colocalization analyses of fluorescence images were conducted using the Coloc2 plugin in ImageJ. To assess the degree of co-occurrence between channels 1 and 2, the Costes method was used for pixel intensity correlation over space. Kendall's tau-b correlation analysis was later used to test the significance of correlation. To avoid any manipulation of fluorescence images, background was not subtracted.

RNA extraction, cDNA synthesis, and RT-PCR: Total RNA was extracted from snap-frozen liver tissues using a TRIzol™ (Invitrogen #15596026) and PureLink RNA Mini Kit (Invitrogen,

#12183018A) combined method. Reverse transcription was carried out with 1 μg of total RNA using the Superscript III First Strand Synthesis system (Invitrogen #18080-051). The synthesized cDNA was used for real-time quantitative PCR (RT-qPCR) with the *Power* SYBR™ Green PCR mix (Applied Biosystems #4367659). Target gene expression abundance was determined by quantitative real-time qRT-PCR using the specific primers Axin2: forward 5'-CGCTTTGATAAGGTCCTGGC-3'; reverse 5'-CTCATGTGAGCCTCCTCTCTTT-3'; *GLUL*: forward 5'-CGTTTTATCTTGCATCGGGT-3'; reverse 5'-CCTCAATGCACTTCAGACCA-3'; c-Met: forward 5'-TGGGCACCGAAAGATAAACCT-3'; reverse 5'-TCGGACTTTGCTAGTGCCTC-3'.

RNA-seq and data analysis: RNA-seq was performed by the UTHSA Genome Sequencing Facility. Approximately 500 ng of total RNA was used for RNA-seq library preparation following the Illumina TruSeq stranded mRNA sample preparation guide. The first step in the workflow involves purifying the poly-A-containing mRNA molecules using poly-T oligo-attached magnetic beads. Following purification, mRNA is fragmented into small pieces using divalent cations under elevated temperature. The cleaved RNA fragments are copied into first strand cDNA using reverse transcriptase and random primers. This is followed by second strand cDNA synthesis using DNA Polymerase I and RNase H. Strand specificity is achieved by replacing dTTP with dUTP in the Second Strand Marking Mix (SMM). These cDNA fragments then go through an end repair process, the addition of a single 'A' base, and then ligation of the adapters. The products were then purified and enriched with PCR to create the final RNA-seq library. After RNA-seq libraries were subjected to a quantification process, they were pooled for cBot amplification and subsequent 50 bp single read sequencing run with the Illumina HiSeq 3000 platform. After the sequencing run, demultiplexing with Bcl2fastq2 was employed to generate the fastq file for each sample. An average of 37 M reads were obtained for this set of samples.

The raw reads were aligned to the reference Mus musculus genome (UCSC mm10) with HISAT2. Genes were annotated and quantified by feature counts, and DEseq was used to identify differentially expressed genes (DEGs). Plots were generated, and the related statistics were performed using R version 3.4.1. Representative RNA-seq results were also validated using RT-PCR.

Gene set enrichment analysis (GSEA) was performed using GSEA v4.2.2 software. Gene expression in human liver cancer patients in TCGA dataset was retrieved from cBioProtal (www.cbioportal.org). DNA methylation in the TCGA dataset was retrieved from MEXPRESS (9). The patient survival data shown in Fig. 7 were retrieved from GSE14520 (10).

Cell growth/proliferation assay: Cells were seeded at 40,000 per well in triplicate wells in 12-well plates. The next day, an initial seeding plate was fixed with 4% paraformaldehyde (PFA) solution in PBS. The remaining plates were washed two times with PBS before receiving the indicated culture media. Cells were cultured for 24, 48, and 72 h and then fixed with 4% PFA. After fixation, the cells were stained with 0.1% crystal violet. The stain was washed three times with double-distilled water and eluted with 10% acetic acid. Relative growth was determined by the optical density of crystal violet staining at 590 nm and normalized to that of the initial cell density. Culture plates were photographed prior to the elution process.

LC-MS: Twenty to forty milligrams of frozen liver tissue samples were weighed and kept in prechilled 2 mL Eppendorf tubes with beads and ground with CryoMill (Retsch NC1216630) at liquid nitrogen temperature and 20 Hz for 2 min. The ground samples were extracted by adding 40 times -20°C 40:40:20 methanol:acetonitrile:water solution *with 0.5% formic acid*, followed by vortexing and centrifugation at 16,000 x g for 10 min at 4°C. Forty-four microliters of 15% NH_4HCO_3 was added to 500 μl of the transferred extract to neutralize the acid. The samples were centrifuged at 16,000 x g for 10 min to remove the protein precipitate. Then, 300 μl of the supernatant was collected into a new tube for LC-MS.

For plasma samples, 50 μl of plasma was transferred into a 1.5 mL tube and extracted by adding 200 μl of ice-cold methanol, followed by vortexing, sitting at -20°C freezer for 20 min, and centrifuging at 16,000 x g for 10 min at 4°C. The supernatant was collected into a new tube, while the pellets were further extracted by adding 1,000 µl 40:40:20 methanol:acetonitrile:water solution, followed by vortexing, sitting on ice for 10 min, and centrifuging at 16,000 x g for 10 min at 4°C. The supernatant was combined and used for LC-MS.

For in vitro experiments, Hep3B cells were seeded in 6-well plates at equal cell number. Cells were washed three times with cold PBS before culturing in media with stable isotope tracers. After tracing, media was aspirated, then quickly overlaid with 0.5 mL of 40:40:20 mixture of methanol:acetonitrile:water with 0.5% formic acid. The plates were incubated on ice for 5 min,

then 25 μl of 15% NH_4HCO_3 was added to neutralize acetic acid. Cells were scraped into microfuge tubes, and centrifuged for 10 min at 15,000 x g at 4°C. The supernatant was collected for LC-MS.

The LC-MS method involved hydrophilic interaction chromatography (HILIC) coupled with electrospray ionization to a Q Exactive PLUS hybrid quadrupole-orbitrap mass spectrometer (Thermo Scientific). LC separation was performed on an XBridge BEH Amide column (150 mm × 2.1 mm, 2.5 μm particle size, Waters, Milford, MA) using a gradient of solvent A (95%/5% H_2O/acetonitrile with 20 mM ammonium acetate and 20 mM ammonium hydroxide, pH 9.4) and solvent B (20%/80% H_2O/acetonitrile with 20 mM ammonium acetate and 20 mM ammonium hydroxide, pH 9.4). The gradient was 0 min, 100% B; 3 min, 100% B; 3.2 min, 90% B; 6.2 min, 90% B; 6.5 min, 80% B; 10.5 min, 80% B; 10.7 min, 70% B; 13.5 min, 70% B; 13.7 min, 45% B; 16 min, 45% B; 16.5 min, 100% B; and 22 min, 100% B. The flow rate was 300 μL/min. The injection volume was 5 μL, and the column temperature was 25°C. The automatic gain control (AGC) target was 3×10^6. The maximum injection time was 50 ms. The scan range was m/z 75-1,000 for general metabolomics and m/z 55-500 for urea measurements. The metabolite features were extracted in MAVEN (11) with the labeled isotope specified and a mass accuracy window of 5 ppm. The isotope natural abundance and tracer isotopic impurity were corrected using AccuCor (12).

Pathway analysis was generated by MetaboAnalyst 5.0. The data were subjected to log transformation and scaling before analysis. The KEGG pathway of Mus musculus was selected as the pathway library.

References:

1. Qiao Y, Xu M, Tao J, Che L, Cigliano A, Monga SP, et al. Oncogenic potential of N-terminal deletion and S45Y mutant beta-catenin in promoting hepatocellular carcinoma development in mice. *BMC Cancer.* 2018;18(1):1093.
2. Chen X, and Calvisi DF. Hydrodynamic transfection for generation of novel mouse models for liver cancer research. *Am J Pathol.* 2014;184(4):912-23.
3. Qiao Y, Wang J, Karagoz E, Liang B, Song X, Shang R, et al. Axis inhibition protein 1 (Axin1) Deletion-Induced Hepatocarcinogenesis Requires Intact beta-Catenin but Not Notch Cascade in Mice. *Hepatology.* 2019;70(6):2003-17.
4. Heindryckx F, Colle I, and Van Vlierberghe H. Experimental mouse models for hepatocellular carcinoma research. *Int J Exp Pathol.* 2009;90(4):367-86.
5. Bakiri L, and Wagner EF. Mouse models for liver cancer. *Mol Oncol.* 2013;7(2):206-23.

6. Sullivan MR, Danai LV, Lewis CA, Chan SH, Gui DY, Kunchok T, et al. Quantification of microenvironmental metabolites in murine cancers reveals determinants of tumor nutrient availability. *Elife.* 2019;8.
7. Poillet-Perez L, Xie X, Zhan L, Yang Y, Sharp DW, Hu ZS, et al. Autophagy maintains tumour growth through circulating arginine. *Nature.* 2018;563(7732):569-73.
8. Nieto R, Calder AG, Anderson SE, and Lobley GE. Method for the determination of 15NH3 enrichment in biological samples by gas chromatography/electron impact ionization mass spectrometry. *J Mass Spectrom.* 1996;31(3):289-94.
9. Koch A, De Meyer T, Jeschke J, and Van Criekinge W. MEXPRESS: visualizing expression, DNA methylation and clinical TCGA data. *BMC Genomics.* 2015;16:636.
10. Roessler S, Jia HL, Budhu A, Forgues M, Ye QH, Lee JS, et al. A unique metastasis gene signature enables prediction of tumor relapse in early-stage hepatocellular carcinoma patients. *Cancer Res.* 2010;70(24):10202-12.
11. Melamud E, Vastag L, and Rabinowitz JD. Metabolomic analysis and visualization engine for LC-MS data. *Anal Chem.* 2010;82(23):9818-26.
12. Su X, Lu W, and Rabinowitz JD. Metabolite Spectral Accuracy on Orbitraps. *Anal Chem.* 2017;89(11):5940-8.
